# Growth hormone (GH)‐transgenic insulin‐like growth factor 1 (IGF1)‐deficient mice allow dissociation of excess GH and IGF1 effects on glomerular and tubular growth

**DOI:** 10.14814/phy2.12709

**Published:** 2016-03-20

**Authors:** Andreas Blutke, Marlon R. Schneider, Eckhard Wolf, Rüdiger Wanke

**Affiliations:** ^1^ Institute of Veterinary Pathology at the Centre for Clinical Veterinary MedicineLudwig‐Maximilians‐University MuenchenMunichGermany; ^2^ Chair for Molecular Animal Breeding and BiotechnologyGene CentreLudwig‐Maximilians‐University MuenchenMunichGermany

**Keywords:** Glomerulosclerosis, kidney, quantitative stereology

## Abstract

Growth hormone (GH)‐transgenic mice with permanently elevated systemic levels of GH and insulin‐like growth factor 1 (IGF1) reproducibly develop renal and glomerular hypertrophy and subsequent progressive glomerulosclerosis, finally leading to terminal renal failure. To dissociate IGF1‐dependent and ‐independent effects of GH excess on renal growth and lesion development in vivo, the kidneys of 75 days old IGF1‐deficient (*I* ^*−/−*^) and of IGF1‐deficient GH‐transgenic mice (*I* ^*−/−*^ */G*), as well as of GH‐transgenic (*G*) and nontransgenic wild‐type control mice (*I* ^*+/+*^) were examined by quantitative stereological and functional analyses. Both *G* and *I* ^*−/−*^ */G* mice developed glomerular hypertrophy, hyperplasia of glomerular mesangial and endothelial cells, podocyte hypertrophy and foot process effacement, albuminuria, and glomerulosclerosis. However, *I* ^*−/−*^ */G* mice exhibited less severe glomerular alterations, as compared to *G* mice. Compared to *I* ^*+/+*^ mice, *G* mice exhibited renal hypertrophy with a significant increase in the number without a change in the size of proximal tubular epithelial (PTE) cells. In contrast, *I* ^*−/−*^ */G* mice did not display significant PTE cell hyperplasia, as compared to *I* ^*−/−*^ mice. These findings indicate that GH excess stimulates glomerular growth and induces lesions progressing to glomerulosclerosis in the absence of IGF1. In contrast, IGF1 represents an important mediator of GH‐dependent proximal tubular growth in GH‐transgenic mice.

## Introduction

During the last decades, a significant role of the growth hormone (GH) and insulin‐like growth factor 1 (IGF1) system in kidney development and function, and in the pathogenesis of chronic kidney diseases (CKD) and their sequelae, has become apparent (Wolf et al. 2000; Schrijvers et al. 2004; Mak et al. 2008; Kumar et al. 2011; Kamenicky et al. 2014; Bach and Hale 2015). In different entities of CKD, including diabetic nephropathy (DN), progressive glomerulosclerosis is the common histopathological feature, and is regarded as the determining mechanism driving the progressive loss of functioning nephrons and subsequent renal scarring, finally leading to terminal renal failure (Klahr et al. 1988; Fogo and Ichikawa 1989, 1991; el Nahas 1989). There is strong evidence for an involvement of the GH/IGF1 axis in induction, maintenance, and progression of glomerular and podocyte hypertrophy and damage, which represent the pathogenetic key lesions triggering development of progressive glomerulosclerosis (Fogo and Ichikawa 1991; Wanke et al. 2001; Kriz 2002; Pavenstadt et al. 2003; Reddy et al. 2007; Kumar et al. 2011). The proceeding enlargement of glomeruli and podocytes induces a characteristic sequence of alterations, finally leading to obsolescence of the glomerulus, atrophy of the corresponding nephron, and subsequent tubulointerstitial lesions (Fogo and Ichikawa 1991; Wiggins 2007). Glomerular hypertrophy is characterized by increased numbers of glomerular mesangial and endothelial cells and is commonly associated with increased matrix deposition in the glomerular mesangium (Schrijvers et al. 2004; Thrailkill et al. 2009). Unlike mesangial and endothelial cells, visceral epithelial glomerular cells (podocytes) are postmitotically fixed and therefore not capable of hyperplastic growth responses (Kriz 1996; Wanke et al. 2001; Wiggins 2007). Along with the progressive enlargement of the glomerular volume, the mean podocyte volume increases and a characteristic sequence of podocyte alterations subsequently develops, including podocyte foot process effacement, impairment of the glomerular filtration barrier, albuminuria, detachment of podocytes from the glomerular basement membrane (GBM), synechia formation between the capsule of Bowman and denuded GBM areas of the glomerular tuft, capillary and extracapillary hyalinosis, collapse of glomerular capillaries, and misdirected filtration into the periglomerular interstitium with subsequent inflammatory and fibrotic tubulointerstitial lesions and atrophy of nephrons (Kretzler et al. 1994; Kriz 1996, 2002, 2012; Kriz et al. 1996; Wanke et al. 2001; Kriz and LeHir 2005; Wiggins 2007). Much of our understanding and knowledge of the pathogenetically relevant processes in development of glomerulosclerosis was gained by studying the nephropathy of GH‐transgenic mice (Doi et al. 1988, 1990; Wanke et al. 1991, 2001; Wolf et al. 1993; Yang et al. 1993; von Waldthausen et al. 2008). GH‐transgenic mice exhibit permanently elevated systemic concentrations of GH and IGF1. They display a typical spectrum of GH excess associated alterations, such as disproportionately stimulated body and organ growth (Wanke et al. 1991, 1992; Le Roith et al. 2001) and diverse characteristic organ lesions (Doi et al. 1990; Wanke et al. 1991, 1992, 2001; Stefaneanu et al. 1993; Wolf et al. 1993; Wolf and Wanke 1997; Dirsch et al. 1998; Miquet et al. 2008; Blutke et al. 2014). In the kidneys, GH‐transgenic mice reproducibly develop the full spectrum of the lesions delineated above, from induction of overall renal growth and glomerular hypertrophy to age‐related development of progressive glomerulosclerosis and tubulointerstitial lesions, finally leading to terminal renal failure (end‐stage renal disease) (Wanke et al. 1991, 2001; Wolf et al. 1993). However, the exact roles of excess GH and of subsequently elevated IGF1 in stimulation of renal and glomerular growth in GH‐transgenic mice are yet not completely clarified. Since GH‐transgenic mice have been and will continue to be used as valuable models enabling the study of diverse aspects of different human nephropathies, including the essential involvement of the GH/IGF1 axis in the development of glomerulosclerotic alterations (Reddy et al. 2007; Kumar et al. 2010, 2011; Grunenwald et al. 2011) and the testing of new therapeutic strategies, it is of crucial importance to further improve the understanding of the contribution of single factors of the GH/IGF1 axis to the pathomechanisms underlying the nephropathy in the model of the GH‐transgenic mouse. Therefore, the present study used a unique collective of IGF1‐deficient GH‐transgenic mice (*I* ^*−/−/*^ *G*) (Blutke et al. 2014) to dissociate IGF1‐dependent and IGF1‐independent excess GH‐stimulated morphologic growth effects on different renal compartments, nephron structures, and cell types in vivo.

## Glossary

### Mouse genotypes

**Table:** 

*I* ^*−/−*^	IGF1‐deficient mice
*I* ^*−/−*^ */G*	IGF1‐deficient, GH‐transgenic mice
*I* ^*+/−*^	Heterozygous IGF1‐deficient mice
*I* ^*+/−*^ */G*	Heterozygous IGF1‐deficient, GH‐transgenic mice
*I* ^*+/+*^	Wild‐type control mice
*G*	GH‐transgenic mice

### Analysis techniques, reagents, and histological stains

**Table:** 

TEM	Transmission electron microscopy
GMA/MMA	Glycolmethacrylate/methylmethacrylate
HE	Hematoxylin and eosin
PAS	Periodic acid Schiff
PASM	Periodic acid silver methenamine

### Abbreviations of anatomical structures used in stereological terms

**Table:** 

Kid	Kidney
OSM	Outer stripe of the medulla
ISM/IZM	Inner stripe and inner zone of medulla
PT	Proximal tubules
OT	Other tubules
PTE	Proximal tubular epithelium
Pod	Podocyte
Glom	Glomerulus
GBM	Glomerular basement membrane
Cap	Capillary

#### Weight and density

**Table:** 

KW	Weight of both kidneys	(mm³)
*ρ*(Kid)	Kidney density	(mg/mm³)

### Morphometrical and quantitative stereological parameters

#### Volume densities (fractional volumes)

**Table:** 

*V*_V(Cortex/Kid)_	Volume density of the renal cortex in the kidneys	v/v
*V*_V(OSM/Kid)_	Volume density of the OSM in the kidneys	v/v
*V*_V(ISM/IZM/Kid)_	Volume density of the ISM/IZM in the kidneys	v/v
*V*_V(Glom/Cortex)_	Volume density of the glomeruli in the renal cortex	v/v
*V*_V(Pod/Glom)_	Volume density of podocytes in the glomeruli	v/v
*V*_V(Cap/Glom)_	Volume density of capillaries in the glomeruli	v/v
*V*_V(Mes/Glom)_	Volume density of the mesangium in the glomeruli	v/v
*V*_V(PT/Cortex+OSM)_	Volume density of PT in the renal cortex and the OSM	v/v
*V*_V(OT/Cortex+OSM)_	Volume density of OT in the renal cortex and the OSM	v/v
*V*_V(PTE/Cortex+OSM)_	Volume density of the PTE in the renal cortex and the OSM	v/v
*f*_s_	Linear tissue shrinkage factor; *f*_s_ = 0.91 for GMA/MMA‐, and *f*_s_ = 0.95 for Epon‐embedded murine kidney tissue	

#### Volumes

**Table:** 

*V*_(Kid)_	Volume of both kidneys	(mm³)
*V*_(Cortex, Kid)_	Volume of the renal cortex in both kidneys	(mm³)
*V*_(OSM, Kid)_	Volume of the OSM in both kidneys	(mm³)
*V*_(Cortex+OSM, Kid)_	Volume of the renal cortex and the OSM in both kidneys	(mm³)
*V*_(ISM/IZM, Kid)_	Volume of the ISM/IZM in both kidneys	(mm³)
*V*_(PT, Cortex+OSM)_	Volume of the PT in the renal cortex and the OSM in both kidneys	(mm³)
*V*_(OT, Cortex+OSM)_	Volume of OT in the renal cortex and the OSM in both kidneys	(mm³)
*V*_(PTE, Cortex+OSM)_	Total PTE volume in the renal cortex and the OSM in both kidneys	(mm³)
*V*_(Glom, Cortex)_	Volume of all glomeruli in the renal cortex in both kidneys	(mm³)
*V*_(Glom‐cap, Kid)_	Volume of all glomerular capillaries in both kidneys	(mm³)
$\overset{¯}{v}$_(Glom)_	Mean glomerular volume	(10³ *μ*m³)
$\overset{¯}{v}$_(Glom)_/BW	Mean glomerular volume relative to body weight	(10^−3^ *μ*m³/g)
$\overset{¯}{v}$_(Glom)_/KW	Mean glomerular volume relative to kidney weight	(*μ*m³/mg)
$\overset{¯}{v}$_(PTE cell, Cortex+OSM)_	Mean volume of PTE cells in the cortex and OSM	(10³ *μ*m³)
$\overset{¯}{v}$_(Pod)_	Mean podocyte volume	(*μ*m³)
$\overset{¯}{v}$_(Cap, Glom)_	Mean capillary volume per glomerulus	(10³ *μ*m³)
$\overset{¯}{v}$_(Mes, Glom)_	Mean mesangial volume per glomerulus	(10³ *μ*m³)
*V*_(Pod, Glom)_	Average volume of the podocytes in one glomerulus	(10³ *μ*m³)

#### Numerical volume densities

**Table:** 

*N*_V(Glom/Kid)_	Numerical volume density of glomeruli in the renal cortex	(n/10^5^ *μ*m³)
*N*_V(PTE cells/Cortex+OSM)_	Numerical density of PTE cells in the renal cortex	(n/10^3^ *μ*m³)
*N*_V(C/Glom)_	Numerical volume density of glomerular cells in the glomeruli	(n/10^5^ *μ*m³)
*N*_V(M‐E/Glom)_	Numerical volume density of mesangial and endothelial cells in the glomeruli	(n/10^5^ *μ*m³)
*N*_V(Pod/Glom)_	Numerical volume density of podocytes in the glomeruli	(n/10^5^ *μ*m³)

#### Total numbers

**Table:** 

*N*_(Glom, Cortex)_	Total number of glomeruli in the cortex of both kidneys
*N*_(PTE cells, Cortex+OSM)_	Total number of PTE cells in the renal cortex and the OSM of both kidneys
*N*_(Pod, Kid)_	Total number of podocytes in all glomeruli of in both kidneys
*N*_(C, Glom)_	Mean number of glomerular cells per glomerulus
*N*_(M‐E, Glom)_	Mean number of glomerular mesangial and endothelial cells per glomerulus
*N*_(Pod, Glom)_	Mean number of podocytes per glomerulus

#### Length densities and lengths

**Table:** 

*L*_V(Cap/Glom)_	Length density of capillaries per glomerulus	(m/mm³)
*L*_(Cap, Glom)_	Average length of the capillaries in one glomerulus	(mm)
*L*_(Glom‐cap, Kid)_	Total length of glomerular capillaries in both kidneys	(m)

### Ultrastructural morphometric glomerular parameters

**Table:** 

FSF (n/mm GBM)	Filtration slit frequency	(n/mm)
*T*_h(GBM)_	The true harmonic mean thickness of the GBM	(nm)
*l*_h(GBM)_	Apparent harmonic mean thickness of the GBM	(nm)

### Equations–General parameters

#### Volume densities

*V*_V(X/Y)_ = *A*_A(X/Y)_ = ∑*A*_(X)_/∑*A*_(Y)_ = ∑Pt_(X)_/∑Pt_(Y)_ = Pt_Pt(X/Y)_

**Table:** 

*V*_V(X/Y)_	Volume density of the structure X in the reference compartment Y
*A*_A(X/Y)_	Area density of the structure X in the reference compartment Y
∑*A*_(X)_/∑*A*_(Y)_	Quotient of the cumulative section area of the structure X in all examined kidney sections per case and the cumulative section area of the reference compartment Y in the same sections
∑Pt_(X)_/∑Pt_(Y)_	Quotient of the total number of points hitting section profiles of the structure X in all examined sections per case and the total number of points hitting the reference compartment Y in the same sections
Pt_Pt(X/Y)_	Point density of the structure X in the reference compartment Y

#### Volumes

*V*_(X, Y)_ = *V*_V(X/Y)_ × *V*_(Y)_

**Table:** 

*V*_(X, Y)_	Total volume of the structure X in the reference compartment Y
*V*_V(X/Y)_	Volume density of the structure X in the reference compartment Y
*V*_(Y)_	Total volume of the reference compartment Y in both kidneys

#### Numerical volume densities

*N*_V(X/Y)_ = (∑Q^*−*^_(X)_/h × ∑A_(Y)_) × *f*_s_³

**Table:** 

*N*_V(X/Y)_	Numerical volume density of elements of the structure X in the reference compartment Y
∑*Q* ^*−*^_(X)_	Cumulative number of all counted elements (*Q* ^−^) of the structure X in all examined disectors per case
h	Disector height, that is, distance between the reference and the “look‐up” section
∑*A*_(Y)_	Cumulative area of the reference compartment sections in all examined disectors per case (for each disector, the mean of the reference compartment section areas in the reference and the “look‐up” section is used for calculation)
h × ∑*A*_(Y)_	Cumulative volume of all disectors examined per case
*f*_s_	Linear tissue shrinkage factor (0.91 for GMA/MMA‐, and 0.95 for Epon‐embedded murine kidney tissue)

#### Numbers

*N*_(X, Y)_ = *N*_V(X/Y)_ × *V*_(Y)_

**Table:** 

*N*_(X, Y)_	Number of individual elements of a distinct structure X in the reference compartment Y
*N*_V(X/Y)_	Numerical volume density of elements of the structure X in the reference compartment Y
*V*_(Y)_	Volume of the reference compartment Y

### Equations–Special parameters

*V*_(Kid)_ = KW/*ρ*_(Kid)_

$\overset{¯}{v}$_(Glom)_ = *V*_(Glom, Cortex)_/*N*_(Glom, Cortex)_ = *V*_V(Glom/Cortex)_/*N*_V(Glom, Cortex)_

$\overset{¯}{v}$_(Mes, Glom)_ = V_V(Mes/Glom)_ × $\overset{¯}{v}$_(Glom)_

$\overset{¯}{v}$_(Pod)_ = *V*_V(Pod/Glom)_/*N*_V(Pod/Glom)_

*V*_(Pod, Glom)_ = $\overset{¯}{v}$_(Glom)_ × *V*_V(Pod/Glom)_

$\overset{¯}{v}$_(Cap, Glom)_ = *V*_V(cap/Glom)_ × $\overset{¯}{v}$_(Glom)_

*V*_(Glom‐cap, Kid)_ = *V*_V(Cap/Glom)_ × *V*_(Glom, Cortex)_

*L*_(Cap, Glom)_ = *L*_V(Cap/Glom)_ × $\overset{¯}{v}$_(Glom)_

*L*_(Glom‐cap, Kid)_ = *L*_(Cap, Glom)_ × *N*_(Glom, Cortex)_

$\overset{¯}{v}$_(PTE cell, Cortex+OSM)_ = *V*_V(PTE cells/Cortex+OSM)_/*N*_V(PTE cells/Cortex+OSM)_

*N*_(X, Glom)_ = *N*_V(X, Glom)_ × $\overset{¯}{v}$_(Glom)_

**Table:** 

*N*_(X, Glom)_	Average number of cells of type X per glomerulus All glomerular cell types, X = C; Mesangial and endothelial glomerular cells, X = Mes‐E; Podocytes, X = Pod

*L*_V(Cap/Glom)_ = 2 × Σ*Q*_(Cap)_/Σ*A*_(Glom)_ × *f*_s_^2^

**Table:** 

*L*_V(Cap/Glom)_	Length density of capillaries per glomerulus
Σ*Q*_(Cap)_	Number of glomerular capillary section profiles per case
Σ*A*_(Glom)_	Cumulative area of all examined glomerular tuft section profiles
*f*_s_	Linear tissue shrinkage factor

Th_(GBM)_ = (8/3π) × (10^6^/M) × lh_(GBM)_

**Table:** 

Th_(GBM)_	The true harmonic mean thickness of the GBM
M	Final print magnification
lh_(GBM)_	Apparent harmonic mean thickness of the GBM lh_(GBM)_ = ∑N° of observations/∑(Midpoints × N° of observations)

## Materials and Methods

### Ethics statement and animal housing

All experiments were approved by the author's institutional committee on animal care, were carried out in accordance with the German Animal Protection Law, and conformed to international guidelines on the ethical use of animals. All efforts were made to minimize the number of animals used and their suffering. All animals investigated in the present study were maintained under specified pathogen‐free conditions in a closed barrier system on a 12:12‐h light–dark cycle and had free access to a standard rodent diet (V1534; Ssniff, Soest, Germany) and tap water.

### Mice

IGF1‐deficient, GH‐transgenic mice (*I* ^*−*/*−*^/*G*) were generated, as described in detail previously (Blutke et al. 2014). Briefly, female heterozygous IGF1‐deficient mice (*I* ^+/*−*^) mice on NMRI outbred background (Powell‐Braxton et al. 1993; Blutke et al. 2014) were mated with male hemizygous GH‐transgenic NMRI mice (*G*), overexpressing bovine GH under the transcriptional control of the phosphoenolpyruvate‐carboxykinase promoter (Wanke et al. 1996; Hoeflich et al. 2002). Male *I* ^+/*−*^/*G* offspring were then mated with female *I* ^+/*−*^ mice to obtain the following six genotypes: *I* ^*−*/*−*^, *I* ^*−*/*−*^/*G*,* I* ^+/*−*^, *I* ^+/*−*^/*G*,* G*, and wild‐type control mice (*I* ^+/+^). Genotyping was performed by PCR, as described previously (Powell‐Braxton et al. 1993; Hoeflich et al. 2001; Blutke et al. 2014). Samples of kidney tissue examined in the present study included specimens derived from mice which had been investigated in a previously published study on body and organ growth and pathology of *I* ^*−*/*−*^/*G* mice (Blutke et al. 2014). IGF1 serum levels, detection of serum GH, and glomerulosclerosis indices of *I* ^*−*/*−*^, *I* ^*−*/*−*^/*G*,* I* ^+/*−*^, *I* ^+/*−*^/*G*,* G*, and *I* ^*+/+*^ mice have as well been reported in this previous study (Blutke et al. 2014).

### Urine analyses

Spot urine samples were taken weekly (from week 4 to 11) between 2:00 and 3:00 pm and immediately stored at −80°C until assayed. Per week, at least five urine samples were collected from mice of each investigated genotype and sex. Urine creatinine concentrations were determined using an automated analyzer technique (Hitachi, Merck, Darmstadt, Germany). For SDS‐PAGE, urine samples were diluted to a creatinine content of 1.5 mg/dL. Urine proteins were temperature denatured (Thermoblock TB1, Biometra, Germany) and separated using a 12% SDS‐PAGE gel (Protean III, Bio‐Rad, Munich, Germany) together with a broad molecular weight standard (Bio‐Rad) and a mouse albumin standard (Biotrend, Cologne, Germany), as described previously (Herbach et al. 2009). Coomassie blue staining of gels was performed according to a standard protocol.

### Body and kidney weights, kidney processing, histology, and electron microscopy

At 11 weeks of age, *I* ^*−*/*−*^ (5/3), *I* ^*−*/*−*^/*G* (3/4), *I* ^+/*−*^ (5/5), *I* ^+/*−*^/*G* (5/5), *I* ^+/+^ (5/5), and *G* (5/5) (*n *= male/female, if not stated differently) mice were sacrificed. After determination of body weight (Blutke et al. 2014), mice were killed by cervicocranial dislocation and immediately perfused with neutrally buffered 2.5% glutaraldehyde solution through the heart, as described previously (Wanke et al. 2001; Herbach et al. 2009). After immersion fixation in 2.5% glutaraldehyde solution, the kidneys were removed carefully, separated from adjacent tissues, blotted dry, and weighed to the nearest weight (mg). The density of the glutaraldehyde‐fixed kidney tissue was determined according to the method described by Scherle (Scherle 1970) and consistently corresponded to 1.05 g/cm³. Next, the kidneys were cut perpendicular to the longitudinal axis (20 ± 5 approximately 1‐mm thick slices in both kidneys), routinely processed and embedded in plastic for light microscopy and transmission electron microscopy (TEM) as described previously (Hermanns et al. 1981; Hoeflich et al. 2002). For TEM, three 1‐mm³ samples of the renal cortex per animal were selected by systematic random sampling (Nyengaard 1999), postfixed in 1% osmium tetroxide, and routinely embedded in Epon resin. Per case, 10 consecutive 0.5‐*μ*m thick semithin sections were cut from each Epon block and stained with toluidine blue O and safranin. For TEM, ultrathin sections (70–80 nm) were cut, stained with uranyl citrate and lead citrate, and examined (EM10, Zeiss, Eching, Germany). The remaining kidney slices were then embedded in plastic, containing hydroxymethylmethacrylate and methylmethacrylate (GMA/MMA, Sigma‐Aldrich Laborchemikalien GmbH, Seelze, Germany), as described previously (Hermanns et al. 1981). For qualitative and quantitative morphological analyses, plastic sections with a nominal section thickness of 1.5 *μ*m were cut on a Reichert‐Jung 2050 rotary microtome (Leica, Wetzlar, Germany) and stained with hematoxylin and eosin, periodic acid Schiff (PAS), and periodic acid silver methenamine (PASM). For counting and sizing of glomeruli, ~140 consecutive sections with a nominal section thickness of 1 *μ*m were additionally cut from each GMA/MMA block per case, using a Microm HM 360 rotary microtome (Microm, Germany). From each section series, every 20th section was systematically randomly selected (6 ± 2 kidney sections per case) and stained with PAS.

### Quantitative stereological analyses

Design‐based stereological methods, such as the physical disector and point counting (Sterio 1984; Howard and Reed 2004) were used for determination of the volumes of distinct renal zones and nephron segments, of the total number of glomeruli, the number and volume of proximal tubular epithelial cells and of different glomerular cell types, and of glomerular capillary volumes and lengths. Plastic resin‐embedded samples of kidney tissue were used for quantitative stereological analyses, and stereological estimates were corrected for embedding‐related tissue shrinkage.

#### Total volumes of the kidney, renal zones, and distinct nephron segments

The kidney volume was obtained by dividing the kidney weight by kidney density. The cross‐sectional areas of the kidney (Kid) and of individual renal zones in the kidney (Kriz and Koepsell 1974; Kriz and Bankir 1988), that is, the cortex, the outer stripe of the medulla (OSM), and the inner stripe of the medulla together with the inner zone of medulla (ISM/IZM) were planimetrically determined in micrographs of PASM‐PAS‐stained GMA/MMA kidney sections (20 ± 5 per case), using a Videoplan^™^ image analysis system (Zeiss‐Kontron, Eching, Germany). The volume fractions of the cortex (*V*_V(Cortex/Kid)_), the OSM (*V*_V(OSM/Kid)_), and the ISM/IZM (*V*_V(ISM/IZM/Kid)_) in the kidney were calculated according to the principle of Delesse (Howard and Reed 2004). The absolute volumes of the individual renal zones (*V*_(Cortex, Kid)_, *V*_(OSM, Kid)_, *V*_(Cortex+OSM, Kid)_, *V*_(ISM/IZM, Kid)_) were calculated by multiplication of the volume fractions of the respective zones in the kidney with the total kidney volume. The fractional volumes of the glomeruli in the cortex (*V*_V(Glom/Cortex)_) of distinct nephron segments within the cortex and OSM, including proximal tubules (*V*_V(PT/Cortex+OSM)_) and other tubules (*V*_V(OT/Cortex+OSM)_), and of the proximal tubular epithelium (PTE) within the cortex and OSM (*V*_V(PTE/Cortex+OSM)_) were determined by point counting (Weibel 1979) (370 ± 89 points per case) in 11 ± 2 systematically randomly selected areas (Nyengaard 1999) of PASM‐PAS‐stained GMA/MMA sections, as principally described earlier (Hoeflich et al. 2002). The absolute volumes of the respective nephron segments (*V*_(PT, Cortex+OSM)_, *V*_(PTE, Cortex+OSM)_, *V*_(Glom, Cortex)_) were calculated from their corresponding volume fraction and the absolute volume of the reference space (cortex, OSM).

#### Mean glomerular volume and total number of glomeruli

The mean glomerular volume ($\overset{¯}{v}$_(Glom)_) and the total number of nephrons (glomeruli) in both kidneys (*N*_(Glom, Cortex)_) were estimated, using the physical disector method (Sterio 1984; Howard and Reed 2004), in combination with systematic point counting, as principally described in previous publications of our group (Hoeflich et al. 2002; Herbach et al. 2009). Per case, 6 ± 2 PAS‐stained serial GMA/MMA sections pairs were systematically randomly selected (nominal section thickness = 1 *μ*m, disector height = 20 *μ*m). Using an automated stereology system (VIS‐Visiopharm Integrator System^™^ Version 3.4.1.0 with newCAST^™^ software, Visiopharm A/S, Denmark), each 35 ± 10 corresponding locations of the renal cortex were then systematically randomly sampled at 40 ×  final magnification in both sections, automatically congruently aligned, and digitally superimposed with unbiased counting frames (344,017 *μ*m² area) and 9 × 9 point test grids. The cross‐sectional area of cortical kidney tissue present within each sampled counting frame (A_(Cortex)_) was determined by point counting. In the subsequent disector analyses, 62 ± 12 *Q* ^*−*^ (glomeruli) were counted per case. The numerical volume density of glomeruli in the renal cortex was calculated as: *N*_V(Glom/Cortex)_ = (∑*Q* ^*−*^_(Glom)_/h × ∑*A*_(Cortex)_) × *f*_s_³ with *f*_s_ = linear tissue shrinkage factor for GMA/MMA‐embedded murine kidney tissue (0.91) (Herbach et al. 2011). *N*_(Glom, Cortex)_ was then calculated as the product of *N*_V(Glom/Cortex)_ and *V*_(Cortex, Kid)_. Subsequently, the mean glomerular volume was estimated as $\overset{¯}{v}$_(Glom)_ = *V*_V(Glom/Cortex)_/*N*_V(Glom, Cortex)_.

To validate the precise disector heights used for the calculation of the numerical volume densities of glomeruli in the kidney, PTE cells in the renal cortex and glomerular cells in the glomeruli, the thicknesses of GMA/MMA and Epon sections were controlled, using an orthogonal resectioning technique, as described previously (Hoeflich et al. 2002). From the GMA/MMA and Epon section series, a section that was not sampled for the disector analyses was randomly selected and re‐embedded in Epon resin, perpendicularly to its original section plane. The thickness of the re‐embedded sections was then measured in image prints of subsequently prepared AzurII/Safranin‐stained semithin Epon sections taken at 1000 × magnification, using a semiautomatic image analysis system (Videoplan^®^, Zeiss‐Kontron, Germany). Per case, four measurements were made. Since the measured mean thickness of re‐embedded Epon sections was 0.501 ± 0.005 *μ*m and 1.037 ± 0.035 *μ*m for GMA/MMA sections, a section thickness 0.5 *μ*m, respectively of 1.0 *μ*m was consistently used for calculation of the disector volumes.

#### Number and volumes of glomerular cells and of PTE cells

The mean volume and total number of PTE cells, the mean numbers of distinct glomerular cell types per glomerulus (C: All glomerular cells; Pod: Podocytes, M‐E: Mesangial and endothelial cells), and the mean podocyte volume were unbiasedly determined, applying the physical disector method (Sterio 1984; Howard and Reed 2004), principally as described above. For this, the numerical volume density of PTE cells in the cortex and of glomerular cells in the glomerulus was unbiasedly estimated in consecutive serial semithin sections of Epon‐embedded cortical kidney tissue samples; per section series, 2–3 pairs of sections with 1.5‐*μ*m distance between these sections, that is, the *n*th and the *n *+* *3rd section from a series cut with a nominal section thickness of 0.5 *μ*m, were systematically randomly sampled (Howard and Reed 2004). Per case, the corresponding profiles of 9 ± 1 systematically randomly sampled glomeruli were photographed at 400 × magnification in both sections of a section pair, using a Leica DFC 320 camera (Leica, Germany) connected to a microscope (Orthoplan, Leitz, Germany). Images including a size ruler were printed and overlaid with a plastic transparency with 576 equally spaced test points. The areas of the glomerular cross‐sections (*A*_(Glom)_) were planimetrically measured, using a Videoplan^TM^ image analysis system (Zeiss‐Kontron, Germany). The volume fraction of podocytes in the glomeruli *V*_V(Pod/Glom)_ was estimated from the fraction of points hitting podocyte section profiles, and points hitting the corresponding reference space, that is, the glomerular cross‐section profile (Howard and Reed 2004). On the average, 559 ± 273 points were counted per case. All glomerular cell nuclei profiles (C, Pod, M‐E) sampled within a glomerular cross‐section in the first (=reference) section, which were not present in the corresponding glomerular section profile in the second (=look‐up) section, were counted (*Q* ^*−*^). The operation was then repeated by interchanging the roles of the look‐up section and the reference section, increasing the efficiency of cell nuclei counting by factor two. On the average, 228 ± 48 glomerular cell (C) nuclei (*Q* ^*−*^) were counted per case (Pod: 69 ± 17; M‐E: 160 ± 41). The numerical volume density of glomerular cells in the glomerulus (*N*_V(C/Glom)_, *N*_V(Pod/Glom)_, *N*_V(M‐E/Glom)_) was then calculated from the number of *Q* ^*−*^ counted per cell type and the respective disector volume, defined by the cumulative areas of analyzed glomerular section profiles and the distance (disector height = 1.5 *μ*m) between the examined section pairs: *N*_V(X/Glom)_ = (∑*Q* ^*−*^_(X)_/h × ∑*A*_(Glom)_) × *f*_s_³ with X = C, or Pod, or M‐E; h = disector height (1.5 *μ*m) and *f*_s_ = linear tissue shrinkage factor for Epon‐embedded murine kidney tissue (0.95) (Herbach et al. 2009). The mean number of cells per glomerulus (*N*_(C,Glom)_) and of podocytes per glomerulus (*N*_(Pod,Glom)_) was calculated by multiplying the numerical volume density of the respective glomerular cells in the glomerulus with the mean glomerular volume (refer to section 2.6.2.2). The mean podocyte volume ($\overset{¯}{v}$_(Pod)_) was calculated dividing *V*_V(Pod/Glom)_ by *N*_V(Pod/Glom)_.

The mean volume ($\overset{¯}{v}$_(PTE cell, Cortex+OSM_) and the total number of PTE cells (*N*_(PTE cells, Cortex+OSM)_) were assessed in an analogous manner. Digital microscopic images (taken at 400 × magnification) of systematically randomly selected fields (11 ± 3 per case) within the sampled disectors were digitally superimposed with an unbiased counting frame (Howard and Reed 2004) of known area (12,543 *μ*m^2^), and a grid of equally spaced test points (68 points per counting frame). The volume density of the PTE in the renal cortex and the OSM (*V*_V(PTE/Cortex+OSM)_) was estimated by point counting, as the fraction of points hitting PTE cell section profiles, and points hitting the renal cortical tissue section area. On the average, 727 ± 331 points were counted per case. The numerical density of PTE cells in the renal cortex (*N*_V(PTE cells/Cortex+OSM)_) was calculated from averagely 66 ± 17 PTE cells (*Q* ^*−*^) counted within the unbiased counting frames of all investigated disectors per case, and the corresponding cumulative disector volumes per case (product of the disector height (1.5 *μ*m) and the cumulative area of the cortical kidney tissue within the counting frames in all examined disectors): *N*_V(PTE cells/Cortex+OSM)_ = (∑*Q* ^*−*^_(PTE cells)_/h × ∑*A*_(Cortex+OSM)_) x *f*_s_³ with h = disector height (1.5 *μ*m) and *f*_s_ = linear tissue shrinkage factor for Epon‐embedded murine kidney tissue (0.95) (Herbach et al. 2009). *N*_(PTE cells, Cortex+OSM)_ was calculated as the product of *N*_V(PTE cells/Cortex+OSM)_ and *V*_(Cortex+OSM, Kid)_. The mean volume of PTE cells ($\overset{¯}{v}$_(PTE cell, Cortex+OSM)_) was obtained by dividing *V*_V(PTE cells/Cortex+OSM)_ by *N*_V(PTE cells/Cortex+OSM)_.

#### Mesangial and glomerular capillary volumes

The volume densities of capillaries, and of the mesangium within the glomeruli (*V*_V(Cap/Glom)_ and *V*_V(Mes/Glom)_) were determined by point counting (fraction of points hitting capillary section profiles, or the mesangial area in PAS‐stained sections, respectively, and points hitting the glomerular section profile) in 36 ± 5 systematically randomly sampled glomerular profiles in PAS‐stained GMA/MMA sections at 200 × magnification. Per case, 420 ± 58 points were counted. The mean mesangial and capillary volumes per glomerulus ($\overset{¯}{v}$_(Mes, Glom)_ and $\overset{¯}{v}$_(Cap, Glom)_) were calculated as the product of the respective numerical volume density in the glomerulus and the mean glomerular volume ($\overset{¯}{v}$_(Glom)_). The total volume of glomerular capillaries in the kidneys was calculated as *V*_(Glom‐cap, Kid)_ = *V*_V(Cap/Glom)_ × *V*_(Glom, Cortex)_.

#### Mean length of the capillaries in a glomerulus

The mean length of the capillaries in a glomerulus (*L*_(Cap, Glom)_) was determined in the same glomerular profiles sampled in the serial semithin Epon sections used for estimation of glomerular cell numbers. The number of glomerular capillary section profiles (Σ*Q*_(Cap)_ = 671 ± 155 per case) present within the examined glomerular section profiles was counted. The cumulative glomerular tuft profile area of all examined glomerular section profiles (Σ*A*_(Glom)_) was measured planimetrically, as described above. The length density of capillaries per glomerulus was calculated as *L*_V(Cap/Glom)_ = 2 × *Q*_A(Cap/Glom)_ × *f*_s_^2^, with *Q*_A(Cap/Glom)_ = Σ*Q*_(Cap)_/Σ*A*_(Glom)_ and *f*_s_ = 0.95. *L*_(Cap, Glom)_ was then obtained as: *L*_(Cap, Glom)_ =** **Lv(Cap/Glom) × $\overset{¯}{v}$(Glom) (Nyengaard 1999; Howard and Reed 2004). The total length of glomerular capillaries in the kidneys (*L*_(Glom‐cap, Kid)_) was calculated as: *L*_(Glom‐cap, Kid)_ = *L*_(Cap, Glom)_ × *N*_(Glom, Kid)_.

#### Glomerular basement membrane (GBM) thickness and filtration slit frequency (FSF)

The thickness of the GBM was determined by the orthogonal intercept method as described earlier (Ramage et al. 2002; El‐Aouni et al. 2006; Herbach et al. 2009). Per case, ultrathin sections of six glomeruli, that were sampled from semithin sections, were prepared for TEM, and peripheral glomerular capillary loops were photographed (7 ± 1 pictures per case) in a predetermined manner (by half turns of the stage handle). Photographs were developed to a final print magnification of 54,324 × , and covered by a transparent 2.5‐cm^2^ grid. Where gridlines transected the GBM, the shortest distance between the endothelial cell membrane and the outer lining of the lamina rara externa underneath the cell membrane of the epithelial foot processes was measured, using a logarithmic ruler template with dimensions and midpoints, as described in detail earlier (Ramage et al. 2002). The true harmonic mean thickness (*T*_h(GBM)_) of the GBM was estimated as: *T*_h(GBM)_ = (8/3π) × (10^6^/M) × l_h(GBM)_, with l_h(GBM)_ (apparent harmonic mean GBM thickness) = ∑ Number of observations/∑ (Midpoints × number of observations), and M = final print magnification. On the average, 46 ± 7 (range: 31–66) intercepts per animal were measured. The FSF was determined in the same electron micrographs, by counting the number of epithelial filtration slits divided by the length of the peripheral capillary wall at the epithelial interface, as described earlier (El‐Aouni et al. 2006). On the average, 162 ± 50 (range: 71–291) filtration slits were counted per animal.

### Statistical analyses

All data are presented as means ± SD. Data were analyzed by one‐way ANOVA with LSD post hoc tests (IBM SPSS Statistics, Version 18), taking the effects of sex and genotype into account. *P *≤* *0.05 were considered significant. To depict effects of GH‐transgene expression on *I* ^*−*/*−*^, *I* ^+/*−*^, and *I* ^+/+^ mice, sex‐matched GH‐transgenic versus non‐GH‐transgenic mice with corresponding *Igf1* genotypes were compared: *I* ^*−*/*−*^ versus *I* ^*−*/*−*^/*G*,* I* ^+/*−*^ versus *I* ^+/*−*^/*G*, and *I* ^+/+^ versus *G*, if not stated otherwise. Gender‐specific differences were analyzed by comparison of male versus female mice of identical genotypes.

## Results

### Body weight, kidney weight, and kidney volume

IGF1‐deficient mice (*I* ^*−/−*^ */G* and *I* ^*−/−*^ mice) displayed approximately 60% lower body and kidney weights and kidney volumes, as compared to mice with intact or heterozygously disrupted *igf1* alleles (Tables 1 and 2). GH‐transgenic mice consistently displayed significantly higher body weights than non‐GH‐transgenic mice with identical *Igf1* status (*G* vs. *I* ^+/+^; *I* ^+/*−*^/*G* vs. *I* ^+/*−*^; and *I* ^*−*/*−*^/*G* vs. *I* ^*−*/*−*^; Table 1). Similarly, *I* ^*−*/*−*^/*G*,* I* ^+/*−*^/*G*, and *G* mice exhibited higher kidney weights and kidney volumes than *I* ^*−*/*−*^, *I* ^+/*−*^, and *I* ^+/+^ mice, respectively. However, the differences of kidney weights and volumes did not reach statistical significance in male *I* ^+/*−*^/*G* versus *I* ^+/*−*^, and in female *I* ^*−*/*−*^/*G* versus *I* ^*−*/*−*^ mice (Tables 1 and 2). Except for male *I* ^+/*−*^/*G* versus *I* ^+/*−*^ mice, the relative kidney weights (% of body weight) did not differ significantly between GH‐transgenic and non‐GH‐transgenic mice of corresponding *igf1* allele status (Table 1).

**Table 1:** Body and kidney weights

Parameter	*n* (m/f)	*I* ^*−/−*^ (5/3)	*I* ^*−/−*^ */G* (3/4)	*I* ^*+/−*^ (5/5)	*I* ^*+/−*^ */G* (5/5)	I^+/+^ (5/5)	*G* (5/5)
Body weight (BW) (g)	m	15.28 ± 1.49^b^	21.50 ± 0.87^b^	33.10 ± 0.83^b^, ^a^	51.47 ± 1.81^b^, ^a^	37.60 ± 1.15^b^	71.86 ± 4.86^b^, ^a^
	f	10.98 ± 1.70^b^	18.20 ± 2.43^b^	26.16 ± 1.42^b^, ^a^	47.47 ± 2.75^b^, ^a^	34.82 ± 3.87^b^	55.79 ± 6.34^b^, ^a^
Kidney weight (KW) (mg)	m	274 ± 52	329 ± 66	718 ± 83^a^	785 ± 133^a^	612 ± 140^b^, ^a^	1182 ± 255^b^, ^a^
	f	160 ± 18	246 ± 14	351 ± 49^b^, ^a^	564 ± 112^b^, ^a^	433 ± 46^b^, ^a^	771 ± 176^b^, ^a^
Relative KW (% of BW)	m	1.8 ± 0.4	1.5 ± 0.3	2.2 ± 0.3^b^, ^a^	1.5 ± 0.2^b^	1.6 ± 0.4^a^	1.7 ± 0.4
	f	1.5 ± 0.1	1.4 ± 0.3	1.3 ± 0.2^a^	1.2 ± 0.2	1.2 ± 0.1^a^	1.4 ± 0.2

Numbers of examined animals (75 days old) are given in brackets. Data are means ± SD. One‐way ANOVA with LSD post hoc test.

a Statistically significant differences (*P* ≤ 0.05) between male and female mice of the identical genotype.

b Statistically significant differences (*P* ≤ 0.05) between sex‐matched *I* ^*−/−*^ versus *I* ^*−/−*^/*G*,* I* ^*+/−*^ versus I^*+/−*^/*G*, and *I* ^*+/+*^ versus *G* mice.

**Table 2:** Kidney volume and quantitative stereological data of distinct renal zones

Parameter	*n* (m/f)	*I* ^*−/−*^ (5/3)	*I* ^*−/−*^ */G* (3/4)	*I* ^*+/−*^ (5/5)	*I* ^*+/−*^ */G* (5/5)	I^+/+^ (5/5)	*G* (5/5)
*V*_Kid_ (mm³)	m	261 ± 49	313 ± 63	683 ± 79^a^	748 ± 127^a^	583 ± 133^b^, ^a^	1125 ± 243^b^, ^a^
	f	153 ± 17	235 ± 14	334 ± 46^b^, ^a^	537 ± 107^b^, ^a^	413 ± 44^b^, ^a^	734 ± 167^b^, ^a^
*V*_V(Cortex/Kid)_	m	0.65 ± 0.02^a^	0.63 ± 0.01	0.64 ± 0.02^b^, ^a^	0.61 ± 0.01^b^	0.61 ± 0.03	0.62 ± 0.03^a^
	f	0.62 ± 0.01^a^	0.65 ± 0.01	0.60 ± 0.02^a^	0.62 ± 0.03	0.60 ± 0.02^b^	0.65 ± 0.02^b^, ^a^
*V*_(Cortex, Kid)_ (mm³)	m	170 ± 31	198 ± 41	438 ± 49^a^	455 ± 84^a^	355 ± 98^b^, ^a^	691 ± 123^b^, ^a^
	f	94 ± 9	153 ± 10	199 ± 30^b^, ^a^	332 ± 54^b^, ^a^	246 ± 23^b^, ^a^	480 ± 115^b^, ^a^
*V*_V(OSM/Kid)_	m	0.22 ± 0.01	0.21 ± 0.01	0.23 ± 0.01	0.25 ± 0.01	0.23 ± 0.02^a^	0.24 ± 0.02
	f	0.22 ± 0.02	0.21 ± 0.01	0.23 ± 0.04	0.24 ± 0.01	0.25 ± 0.02^b^, ^a^	0.22 ± <0.00^b^
*V*_(OSM, Kid)_ (mm³)	m	57 ± 11	66 ± 16	157 ± 21^a^	188 ± 29^a^	134 ± 32^b^	273 ± 82^b^, ^a^
	f	34 ± 7	50 ± 5	77 ± 15^b^, ^a^	130 ± 29^b^, ^a^	105 ± 17^b^	160 ± 35^b^, ^a^
*V*_V(ISM&IZ/Kid)_	m	0.13 ± <0.00	0.16 ± 0.01	0.13 ± 0.02^a^	0.14 ± 0.01	0.16 ± 0.04	0.14 ± 0.03
	f	0.16 ± 0.01	0.14 ± 0.03	0.17 ± 0.02^b^, ^a^	0.14 ± 0.02^b^	0.15 ± 0.01	0.13 ± 0.02
*V*_(ISM&IZ, Kid)_ (mm³)	m	34 ± 6	48 ± 7	89 ± 20^a^	105 ± 15^a^	93 ± 15	162 ± 50
	f	24 ± 1	32 ± 6	58 ± 10^a^	76 ± 25^a^	61 ± 7	95 ± 22

Numbers of examined animals are given in brackets. Data are means ± SD. One‐way ANOVA with LSD post hoc test.

a Statistically significant differences (*P* ≤ 0.05) between male and female mice of the identical genotype.

b Statistically significant differences (*P* ≤ 0.05) between sex‐matched *I* ^*−/−*^ versus *I* ^*−/−*^/*G*,* I* ^*+/−*^ versus I^*+/−*^/*G*, and *I* ^*+/+*^ versus *G* mice.

### Histological and ultrastructural findings

**Figure 1:**
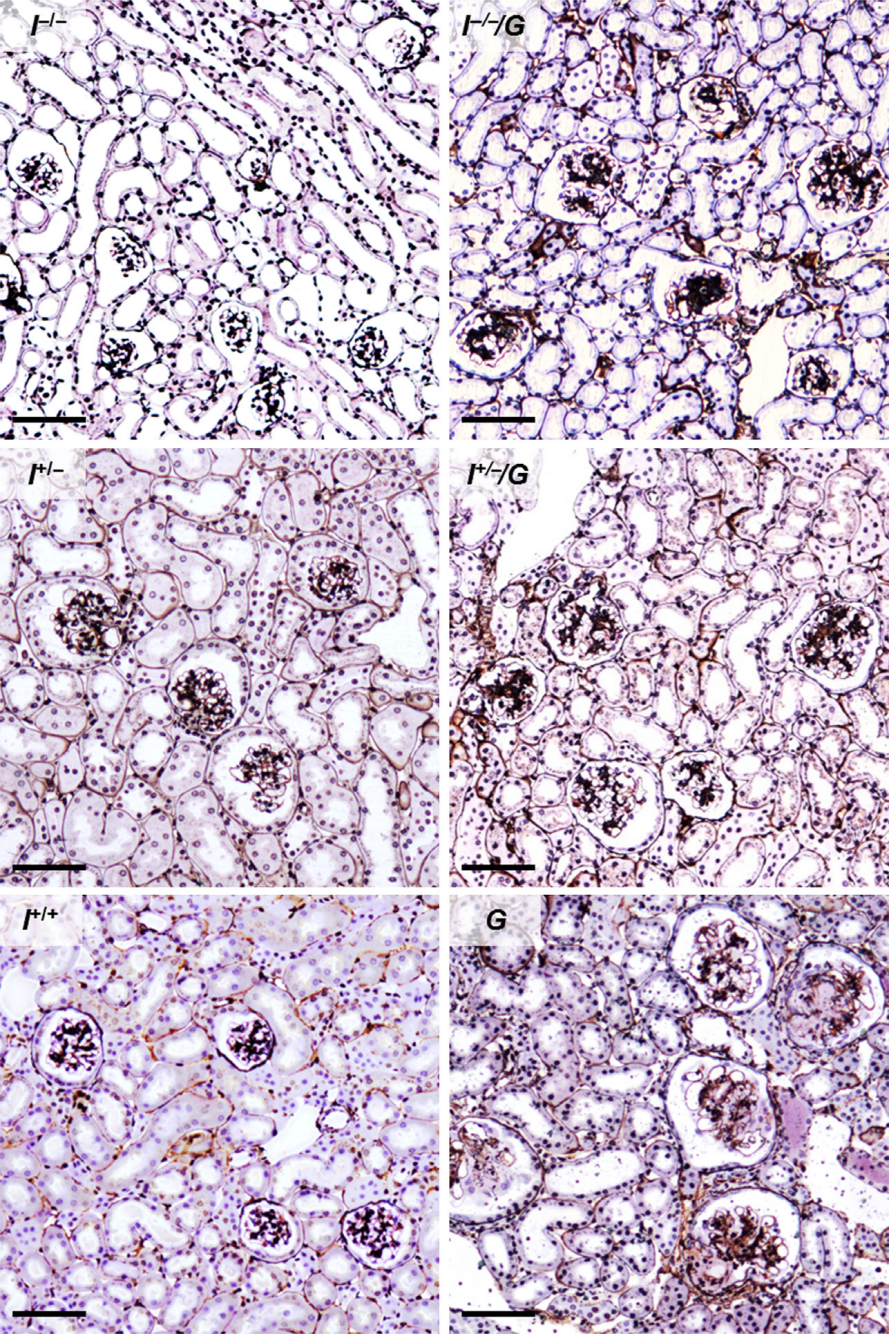
Kidney histology. *I* ^*−*/*−*^/*G*,* I* ^+/*−*^/*G*, and *G* mice display glomerulosclerosis and larger glomerular section profile areas (glomerular hypertrophy), as compared to *I* ^*−*/*−*^, *I* ^+/*−*^, and *I* ^*+/+*^ mice. Male mice at 11 weeks of age. Silver‐PAS staining, GMA/MMA sections. Bars = 50 *μ*m.

**Figure 2:**
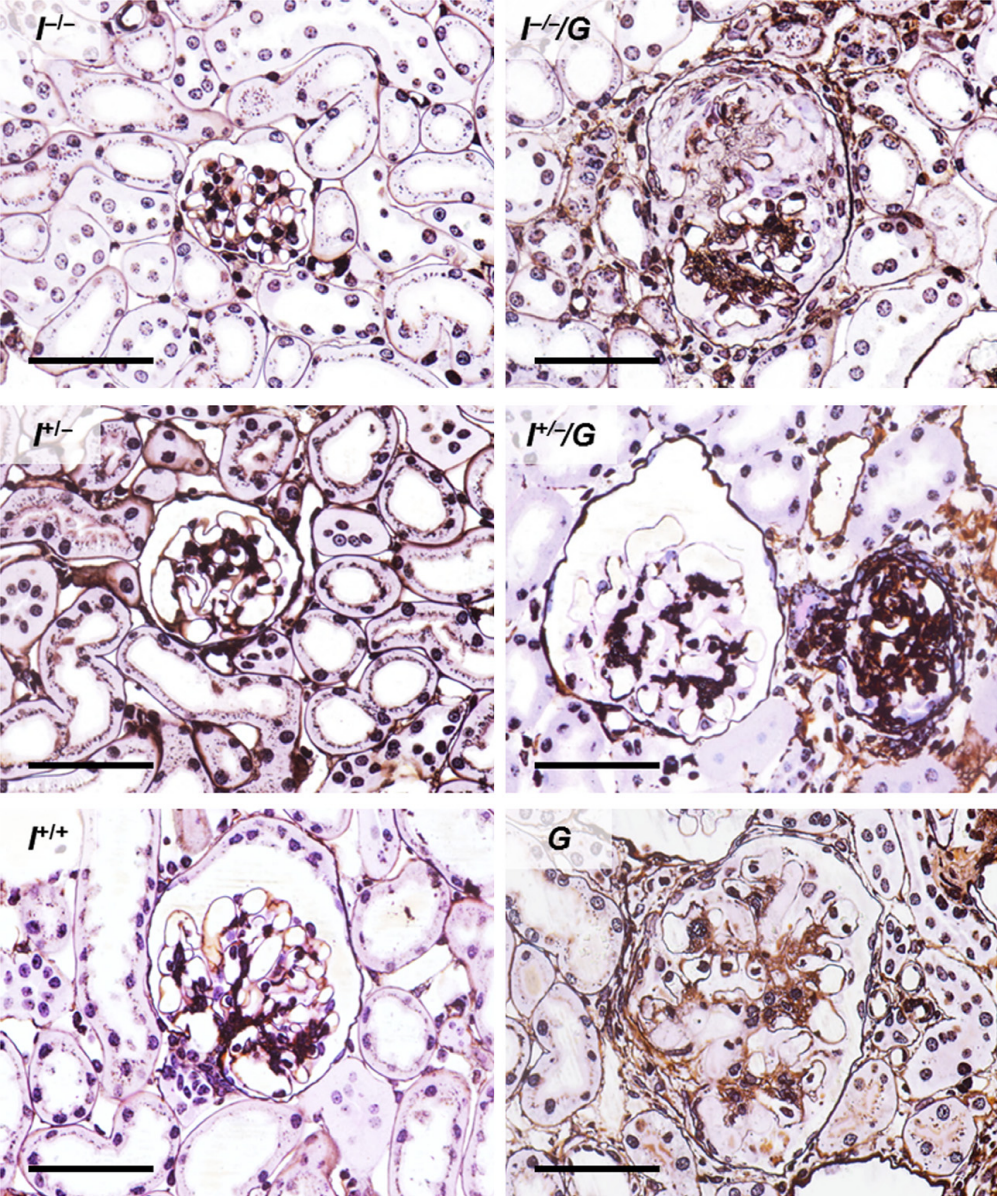
Glomerular histology. *I* ^*−*/*−*^/*G*,* I* ^+/*−*^/*G*, and *G* mice display glomerulosclerosis with glomerular mesangial expansion and matrix accumulation, capillary hyalinosis, and crescent formation. Note the larger glomerular section profile areas in *I* ^*−*/*−*^/*G*, I^+/*−*^/*G*, and *G* mice, as compared to *I* ^*−*/*−*^, *I* ^+/*−*^, and *I* ^*+/+*^ mice (glomerular hypertrophy). Female mice at 11 weeks of age. Silver‐PAS staining, GMA/MMA sections. Bars = 50 *μ*m.

**Figure 3:**
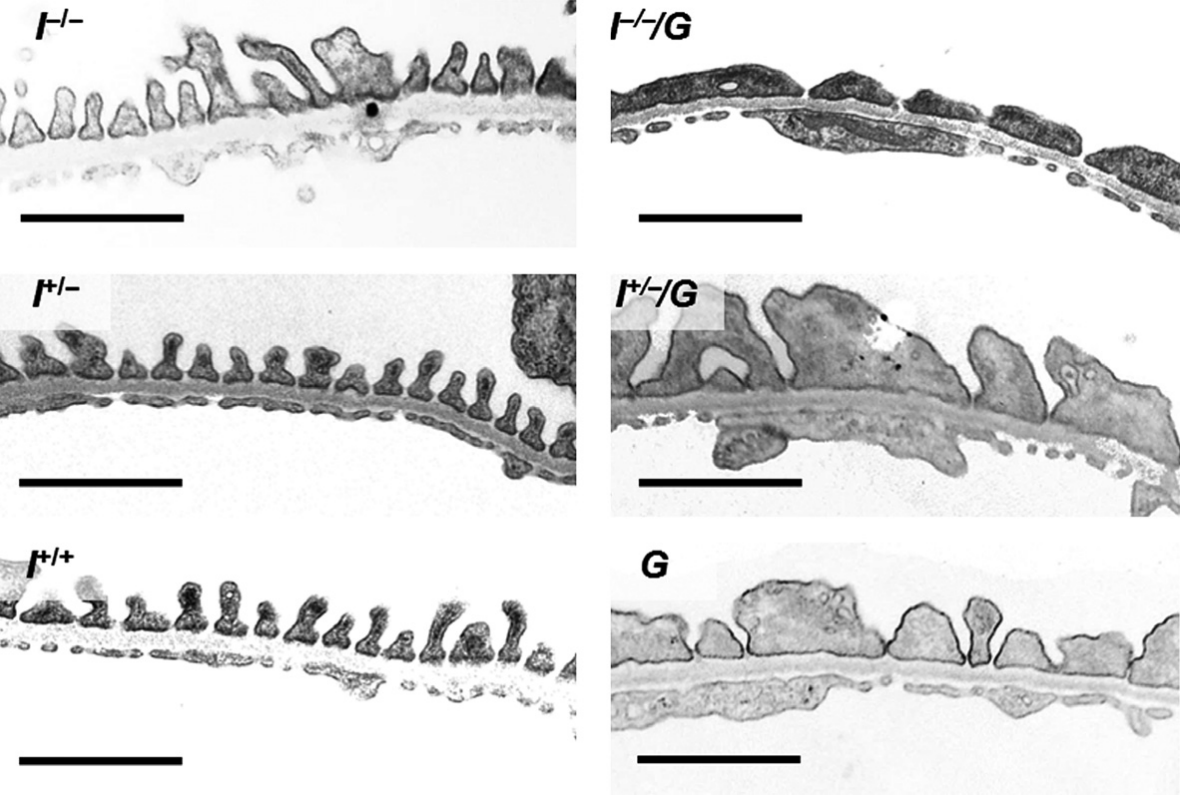
Transmission electron microscopy of peripheral glomerular capillary walls. Mice of GH‐transgenic genotypes (*I* ^*−*/*−*^/*G*, I^+/*−*^/*G*, and *G*) consistently exhibit podocyte foot process effacement and reduced filtration slit frequency, as compared to mice of non‐GH‐transgenic genotypes (*I* ^*−*/*−*^, *I* ^+/*−*^, and *I* ^*+/+*^). Male mice at 11 weeks of age. Bars = 500 nm.

*I* ^*−*/*−*^ mice displayed remarkably smaller glomerular profiles than mice of all other genotypes. In contrast, the glomerular profiles of GH‐transgenic mice (*I* ^*−*/*−*^/*G*,* I* ^+/*−*^/*G* and *G* mice) consistently appeared greatly enlarged, as compared to non‐GH‐transgenic mice with the same *Igf1* status (*I* ^*−*/*−*^, *I* ^+/*−*^, and *I* ^+/+^ mice) (Fig. 1). Histopathological examination revealed glomerulosclerotic lesions present in all GH‐transgenic genotypes (*I* ^*−*/*−*^/*G*,* I* ^+/*−*^/*G*, and *G* mice), comprising glomerular mesangial expansion, matrix accumulation and hypercellularity, multifocal capillary hyalinosis, capillary collapse, formation of synechiae between the glomerular tuft and the capsule of Bowman, crescent formation (Figs. 1 and 2), as well as tubulo‐interstitial lesions, such as mild inflammatory cell infiltration, interstitial fibrosis, and multifocal tubular atrophy, along with signs of proteinuria, that is, accumulation of protein droplets in proximal tubular epithelial cells, and tubular protein casts. These alterations were most marked in G mice, followed by *I* ^+/*−*^/*G*, and *I* ^*−*/*−*^/*G* mice. In contrast, mice of non‐GH‐transgenic genotypes (*I* ^*−*/*−*^, *I* ^+/*−*^, and *I* ^+/+^) did not show glomerulosclerotic alterations. Ultrastructurally, the glomerular podocytes of mice of GH‐transgenic genotypes (*I* ^*−*/*−*^/*G*,* I* ^+/*−*^ */G*, and *G*) appeared enlarged, as compared to non‐GH‐transgenic mice and displayed podocyte foot process effacement (Fig. 3).

### Urine analyses

**Figure 4:**
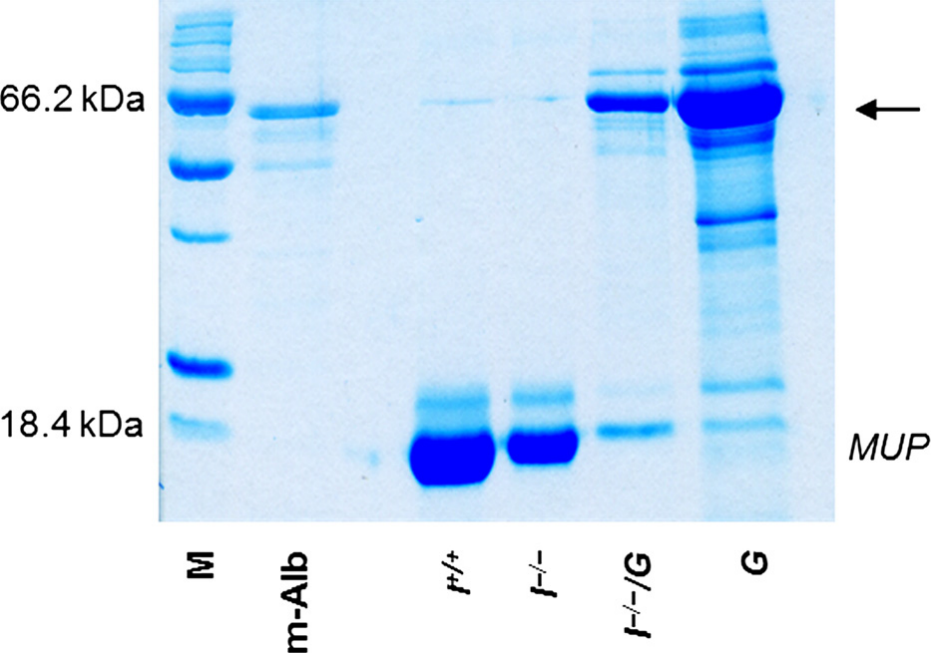
SDS‐PAGE urine protein analysis. Representative gel of 11‐week‐old male mice. Spot urine samples were diluted to identical creatinine concentrations (3 mg/dL) and run on a 12% SDS‐PAGE. Coomassie staining. The *I* ^*−*/*−*^/*G* and *G* mice display albuminuria (bands at approx. 63 kDa, arrow). Also note the drastically reduced intensity of the major urinary protein (MUP) bands at approximately 18 kDa in urine samples of male *I* ^*−*/*−*^/*G* and *G* mice, as compared to male *I* ^*−*/*−*^ and *I* ^*+/+*^ mice.

Mice of all GH‐transgenic genotypes (*I* ^*−/−*^ */G*,* I* ^*+/−*^ */G*, and *G*) demonstrated albuminuria from approximately the fifth week of age onward. The areas and staining intensities of albumin bands (~64 kDa) in SDS‐PAGE‐based urine protein analyses were markedly increased in *G* mice, as compared to *I* ^*−/−*^ */G* mice. Typically for male GH‐overexpressing mice (Norstedt and Palmiter 1984), male GH‐transgenic mice of all examined *Igf1* genotypes (*I* ^*−/−*^ */G*,* I* ^*+/−*^ */G*, and *G*) consistently displayed drastically reduced major urinary protein (MUP) bands (<20 kDa), as compared to male non‐GH‐transgenic mice (Fig. 4).

### Quantitative morphological findings

#### Volumes of distinct renal zones and nephron segments

In GH‐transgenic mice versus non‐GH‐transgenic mice of the identical *Igf1* status (*G* vs. *I* ^*+/+*^, *I* ^*+/−*^ */G* vs. *I* ^*+/−*^, and *I* ^*−/−*^ */G* vs. *I* ^*−/−*^), the volume fractions of the cortex (V_V(Cortex/Kid)_), of the outer stripe of the medulla (*V*_V(OSM/Kid)_), and of the inner stripe of the medulla together with the inner zone of medulla (*V*_V(ISM/IZM/Kid)_) in the kidneys showed only minor, slightly varying (3–8%) differences (Table 2). The absolute volumes of the cortex (*V*_(Cortex, Kid)_) and of the outer stripe of the medulla (*V*_(OSM, Kid)_), however, were significantly increased in *G* versus *I* ^*+/+*^ mice, and in female *I* ^*+/−*^ */G* versus *I* ^*+/−*^ mice, but not in *I* ^*−/−*^ */G* versus *I* ^*−/−*^ mice (Table 2). Similarly, the total volumes of proximal tubules, and of other tubules in the renal cortex and the OSM (*V*_(PT, Cortex+OSM)_ and *V*_(OT, Cortex+OSM)_) were significantly increased in *G* versus *I* ^*+/+*^, and female *I* ^*+/−*^ */G* versus *I* ^*+/−*^ mice as well, but not in *I* ^*−/−*^ */G* versus *I* ^*−/−*^ mice (Table 5). In contrast, the total volumes of the glomeruli in the kidneys (V_(Glom, Cortex)_) were significantly increased in all GH‐transgenic mice versus non‐GH‐transgenic mice of the identical *Igf1* status (Table 3).

**Table 3:** Quantitative stereological data of glomeruli

Parameter	*n* (m/f)	*I* ^*−/−*^ 5/3	*I* ^*−/−*^ */G* 3/4	*I* ^*+/−*^ 4/4	*I* ^*+/−*^ */G* 4/4	I^+/+^ 4/4	*G* 4/4
*V*_V(Glom/Cortex)_	m	0.021 ± 0.006^b^	0.049 ± 0.002^b^	0.020 ± 0.002^b^, ^a^	0.051 ± 0.016^b^	0.025 ± 0.003^b^	0.043 ± 0.003^b^
	f	0.024 ± 0.005^b^	0.053 ± 0.008^b^	0.031 ± 0.004^b^, ^a^	0.050 ± 0.005^b^	0.033 ± 0.002	0.041 ± 0.004
*V*_(Glom, Cortex)_ (mm³)	m	3.5 ± 0.5^b^	9.7 ± 1.8^b^	8.3 ± 1.5^b^	22.5 ± 3.9^b^, ^a^	7.8 ± 0.8^b^	30.0 ± 4.0^b^, ^a^
	f	2.3 ± 0.6^b^	8.2 ± 1.5^b^	6.8 ± 1.1^b^	16.3 ± 1.5^b^, ^a^	8.1 ± 0.3^b^	20.9 ± 5.7^b^, ^a^
$\overset{¯}{v}$_(Glom)_/BW (*μ*m³ × 10^*−*3^/g)	m	9.1 ± 1.6^b^	18.8 ± 0.9^b^	7.5 ± 1.3^b^	13.2 ± 1.0^b^	6.4 ± 1.2^b^	12.5 ± 1.9^b^
	f	9.6 ± 1.5^b^	19.8 ± 5.8^b^	8.0 ± 1.6^b^	11.7 ± 1.2^b^	6.9 ± 0.6^b^	10.8 ± 2.2^b^
$\overset{¯}{v}$_(Glom)_/KW (*μ*m³/mg)	m	542 ± 246^b^	1253 ± 154^b^	355 ± 52^b^	885 ± 185^b^	442 ± 84^b^	759 ± 157^b^
	f	665 ± 163^b^	1451 ± 382^b^	565 ± 72^b^	1041 ± 212^b^	565 ± 74	768 ± 143
*N*_V(Glom/Cortex)_ (n/mm³)	m	154 ± 27^b^, ^a^	121 ± 13^b^, ^a^	80 ± 4^a^	76 ± 20	104 ± 7^b^	47 ± 5^b^
	f	231 ± 26^b^, ^a^	158 ± 44^b^, ^a^	146 ± 11^b^, ^a^	91 ± 15^b^	132 ± 24^b^	66 ± 7^b^
*N*_(Glom, Cortex)_	m	25,967 ± 5502	23,694 ± 2652	33,648 ± 2786	33,337 ± 3677	32,342 ± 2366	33,138 ± 4375
	f	21,794 ± 3599	24,230 ± 7391	31,871 ± 2973	28,769 ± 3015	32,716 ± 4213	33,555 ± 7815

Numbers of examined animals are given in brackets. Data are means ± SD. One‐way ANOVA with LSD post hoc test.

a Statistically significant differences (*P* ≤ 0.05) between male and female mice of the identical genotype.

b Statistically significant differences (*P* ≤ 0.05) between sex‐matched *I* ^*−/−*^ versus *I* ^*−/−*^/*G*,* I* ^*+/−*^ versus I^*+/−*^/*G*, and *I* ^*+/+*^ versus *G* mice.

#### Total number of glomeruli and mean glomerular volume

**Figure 5:**
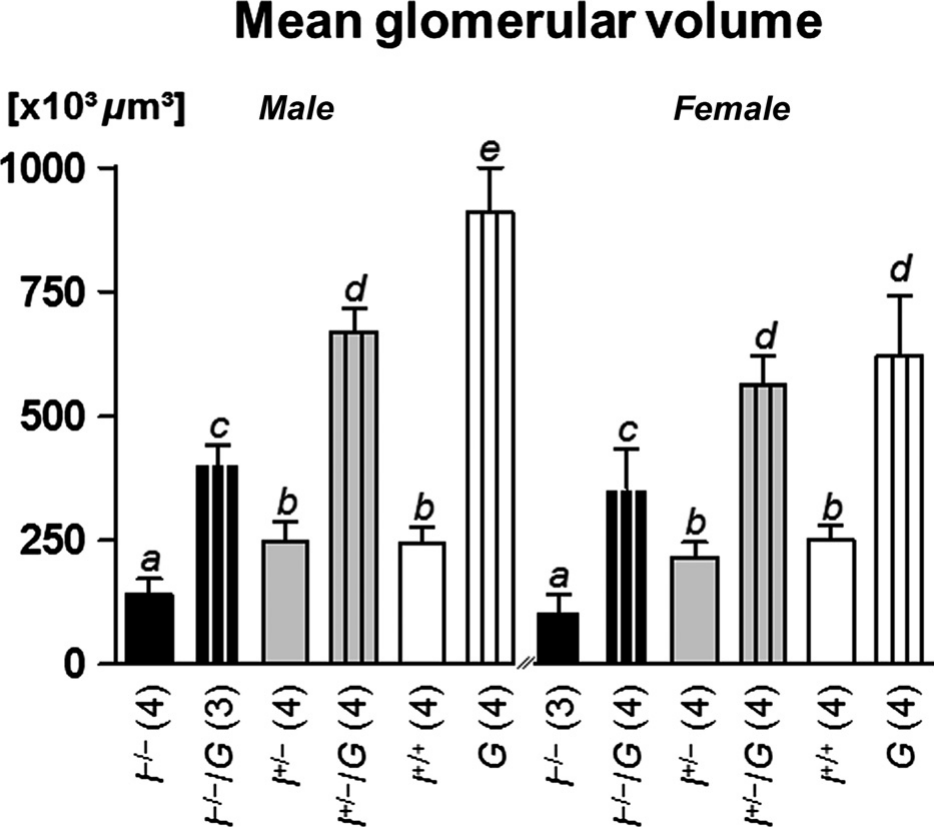
Mean glomerular volume at 11 weeks of age. *I* ^*−*/*−*^/*G*, I^+/*−*^/*G*, and *G* mice display significantly higher mean glomerular volumes, as compared to *I* ^*−*/*−*^, *I* ^+/*−*^, and *I* ^*+/+*^ mice. Data are means ± SD. One‐way ANOVA with LSD post hoc test. Statistically significant differences (*P* ≤ 0.05) are indicated by different superscripts. The numbers of examined animals are given in brackets.

GH‐transgenic mice displayed significantly lower numerical volume densities of glomeruli in the renal cortex (*N*_V(Glom/Cortex)_) than non‐GH‐transgenic mice of identical *Igf1* status, except for *I* ^*+/−*^ */G* versus *I* ^*+/−*^ male mice. The total numbers of glomeruli in the kidneys (*N*_(Glom, Cortex)_), however, did not differ significantly between *I* ^*−/−*^ */G* versus *I* ^*−/−*^, *I* ^*+/−*^ */G* versus *I* ^*+/−*^, and *G* versus *I* ^*+/+*^ mice (Table 3). However, corresponding to their significantly smaller kidney and cortex volumes (Table 2), the total numbers of glomeruli in IGF1‐deficient (*I* ^*−/−*^ */G* and *I* ^*−/−*^) mice were approximately 25% lower than in mice with intact (*I* ^*+/+*^ and *G* mice), or heterozygously disrupted *igf1* alleles (*I* ^*+/−*^ and *I* ^*+/−*^ */G* mice). The mean glomerular volume $\overset{¯}{v}$_(Glom)_, as well as the mean glomerular volume related to body weight ($\overset{¯}{v}$_(Glom)_/BW) was significantly increased in mice of all GH‐overexpressing genotypes, as compared to non‐GH‐transgenic mice of identical *Igf1* status (Table 3, Fig. 5). On the average, *I* ^*−/−*^ */G*,* I* ^*+/−*^ */G*, and *G* mice displayed a 2.7‐fold higher mean glomerular volume, as compared to *I* ^*−/−*^, *I* ^*+/−*^, and *I* ^*+/+*^ mice. Except for female *G* versus *I* ^*+/+*^ mice, also the mean glomerular volume related to kidney weight ($\overset{¯}{v}$_(Glom)_/KW) was significantly increased in GH‐transgenic mice, as compared to non‐GH‐transgenic mice of identical *Igf1* status (Table 3).

#### Mean mesangial and capillary volumes and mean capillary lengths per glomerulus and total glomerular capillary lengths and volumes in the kidneys

Mice of all GH‐transgenic genotypes (*I* ^*−/−*^ */G*,* I* ^*+/−*^ */G*, and *G*) consistently displayed significantly higher volume densities of the mesangium in the glomeruli (*V*_V(Mes/Glom)_), as well as significantly higher mean mesangium volumes per glomerulus ($\overset{¯}{v}$_(Mes, Glom)_), as compared to non‐GH‐transgenic mice of identical *Igf1* status (*I* ^*−/−*^, *I* ^*+/−*^ and *I* ^*+/+*^, respectively). The capillary volume per glomerulus (*V*_(Cap, Glom)_), the total volume of the glomerular capillaries in the kidneys (*V*_(Glom‐cap, Kid)_), the length density of capillaries in the glomeruli (*L*_V(Cap/Glom)_), the mean length of the capillaries per glomerulus (*L*_(Cap, Glom)_), and the total length of the glomerular capillaries in the kidneys (*L*_(Glom‐cap, Kid)_) were also significantly increased in *I* ^*−/−*^ */G* versus *I* ^*−/−*^, *I* ^*+/−*^ */G* versus *I* ^*+/−*^ *,* and *G* versus *I* ^*+/+*^ mice (Table 4).

**Table 4:** Quantitative stereology of glomerular subcompartments

Parameter	*n* (m/f)	*I* ^*−/−*^ (4/3)	*I* ^*−/−*^ */G* (3/4)	*I* ^*+/−*^ (4/4)	*I* ^*+/−*^ */G* (4/4)	*I* ^*+/+*^ (4/4)	*G* (4/4)
*V*_V(Mes/Glom)_	m	0.10 ± 0.03^b^	0.25 ± 0.05^b^	0.11 ± 0.03^b^	0.23 ± 0.07^b^	0.11 ± 0.03^b^	0.25 ± 0.06^b^
	f	0.09 ± 0.02^b^	0.21 ± 0.07^b^	0.12 ± 0.03^b^	0.21 ± 0.04^b^	0.09 ± 0.01^b^	0.23 ± 0.05^b^
$\overset{¯}{v}$_(Mes/Glom)_ (×10³ *μ*m³)	m	15 ± 9^b^	99 ± 15^b^	25 ± 8^b^	155 ± 54^b^	25 ± 6^b^	227 ± 64^b^, ^a^
	f	9 ± 4^b^	75 ± 23^b^	25 ± 5^b^	120 ± 24^b^	24 ± 5^b^	143 ± 37^b^, ^a^
*V*_V(Cap/Glom)_	m	0.63 ± 0.03^b^	0.51 ± 0.05^b^	0.64 ± 0.02^b^, ^a^	0.44 ± 0.03^b^	0.59 ± 0.07^b^	0.46 ± 0.04^b^, ^a^
	f	0.58 ± 0.04^b^	0.44 ± 0.07^b^	0.58 ± 0.03^a^	0.53 ± 0.03	0.58 ± 0.05	0.56 ± 0.06^a^
*V*_(Cap, Glom)_ (×10³ *μ*m³)	m	88 ± 22^b^	209 ± 36^b^	153 ± 33^b^	296 ± 10^b^	144 ± 30^b^	418 ± 38^b^, ^a^
	f	62 ± 19^b^	157 ± 48^b^	125 ± 26^b^	297 ± 50^b^	147 ± 28^b^	346 ± 59^b^, ^a^
*V*_(Glom‐cap, Kid)_ (mm³)	m	2.1 ± 0.2^b^	5.0 ± 1.4^b^	5.0 ± 1.0^b^	9.9 ± 1.3^b^	4.5 ± 0.8^b^	14.1 ± 1.4^b^
	f	1.3 ± 0.3^b^	3.5 ± 0.3^b^	4.4 ± 1.1^b^	8.6 ± 1.1^b^	4.7 ± 0.4^b^	11.5 ± 2.7^b^
*L*_V(Cap/Glom)_ (m/mm³)	m	11.93 ± 0.26^b^	7.10 ± 1.19^b^	12.41 ± 1.61^b^	7.35 ± 1.30^b^	11.83 ± 1.28^b^	5.98 ± 0.47^b^, ^a^
	f	12.30 ± 0.40^b^	8.36 ± 1.54^b^	11.86 ± 0.61^b^	7.90 ± 0.86^b^	11.12 ± 1.02^b^	8.94 ± 1.97^b^, ^a^
*L*_(Cap, Glom)_ (mm)	m	1.67 ± 0.46^b^	2.87 ± 0.50^b^	2.93 ± 0.28^b^	4.91 ± 0.78^b^	2.87 ± 0.57^b^	5.43 ± 0.54^b^
	f	1.31 ± 0.37^b^	2.98 ± 1.02^b^	2.53 ± 0.24^b^	4.41 ± 0.19^b^	2.81 ± 0.57^b^	5.59 ± 1.66^b^
*L*_(Glom‐cap, Kid)_ (m)	m	38.7 ± 4.4^b^	68.1 ± 16.1^b^	95.6 ± 13.7^b^	162.6 ± 25.9^b^, ^a^	92.1 ± 12.8^b^	180.6 ± 35.0^b^
	f	28.4 ± 7.2^b^	66.9 ± 6.5^b^	80.6 ± 9.8^b^	126.9 ± 16.4^b^, ^a^	90.1 ± 9.7^b^	181.5 ± 49.3^b^
*T*_h(GBM)_ (nm)	m (*n* = 3)	157 ± 5	160 ± 7	152 ± 7	155 ± 6	164 ± 6	164 ± 5
	f (*n* = 3)	155 ± 5	165 ± 10	153 ± 6	156 ± 6	165 ± 18	158 ± 10
*N*v_(Cells/Glom)_ (n/10^5^ *μ*m³)	m	131 ± 46^b^	91 ± 20^b^	116 ± 11^b^	69 ± 9^b^	120 ± 22^b^	62 ± 4^b^
	f	186 ± 24^b^	110 ± 19^b^	124 ± 11^b^	65 ± 5^b^	107 ± 11^b^	72 ± 8^b^
*N*v_(Ec&Mc/Glom)_ (n/10^5^ *μ*m³)	m	83 ± 29^a^	67 ± 14	78 ± 8^b^	51 ± 6^b^	80 ± 17^b^	49 ± 3^b^
	f	116 ± 14^b^, ^a^	80 ± 10^b^	80 ± 10^b^	47 ± 4^b^	67 ± 6	56 ± 5
*N*v_(Pod/Glom)_ (n/10^5^ *μ*m³)	m	49 ± 18^b^, ^a^	24 ± 6^b^	38 ± 4^b^	18 ± 3^b^	40 ± 6^b^	14 ± 2^b^
	f	69 ± 10^b^, ^a^	30 ± 11^b^	43 ± 2^b^	17 ± 1^b^	40 ± 4^b^	17 ± 4^b^
*V*v_(Pod/Glom)_	m	0.12 ± 0.04^a^	0.12 ± 0.02	0.13 ± 0.02^b^	0.09 ± 0.01^b^	0.14 ± 0.01	0.15 ± 0.02
	f	0.16 ± 0.05^a^	0.14 ± 0.01	0.14 ± 0.02	0.11 ± 0.01	0.13 ± 0.01	0.12 ± 0.03
*V*_(Pod, Glom)_ (10³ *μ*m³)	m	18 ± 5^b^	49 ± 5^b^	34 ± 6^b^	58 ± 7^b^	33 ± 8^b^	132 ± 19^b^, ^a^
	f	17 ± 4^b^	50 ± 15^b^	29 ± 6^b^	62 ± 13^b^	31 ± 3^b^	75 ± 17^b^, ^a^

Numbers of examined animals are given in brackets. Data are means ± SD. One‐way ANOVA with LSD post hoc test.

a Statistically significant differences (*P* ≤ 0.05) between male and female mice of the identical genotype.

b Statistically significant differences (*P* ≤ 0.05) between sex‐matched *I* ^*−/−*^ versus *I* ^*−/−*^/*G*,* I* ^*+/−*^ versus I^*+/−*^/*G*, and *I* ^*+/+*^ versus *G* mice.

#### Number of cells per glomerulus and mean podocyte volumes

**Figure 6:**
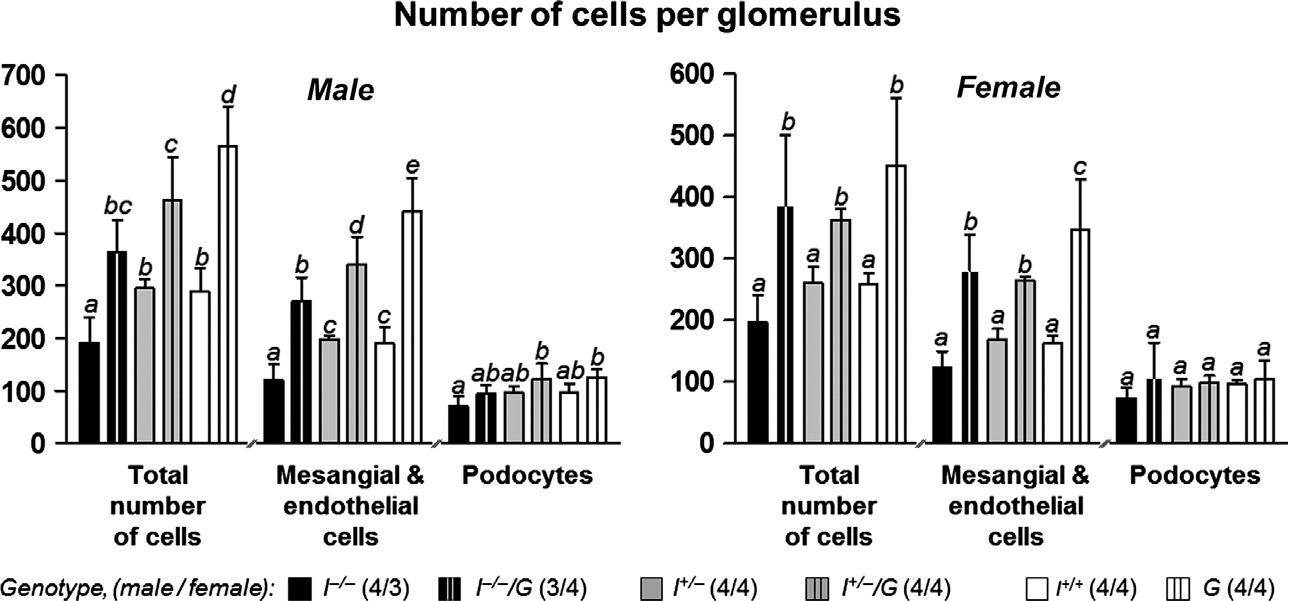
Number of cells per glomerulus of 11‐week‐old mice. The total glomerular cell numbers, as well as the numbers of mesangial and endothelial glomerular cells per glomerulus are significantly higher in *I* ^*−*/*−*^ versus *I* ^*−*/*−*^/*G*,* I* ^+/*−*^ versus I^+/*−*^/*G*, and *I* ^*+/+*^ versus *G* mice. In contrast, the number of podocytes per glomerulus does not significantly differ between GH‐transgenic mice and non‐GH‐transgenic mice with identical *Igf1* status. Data are means ± SD. One‐way ANOVA with LSD post hoc test. Statistically significant differences (*P* ≤ 0.05) are indicated by different superscripts. The numbers of examined animals are given in brackets.

**Figure 7:**
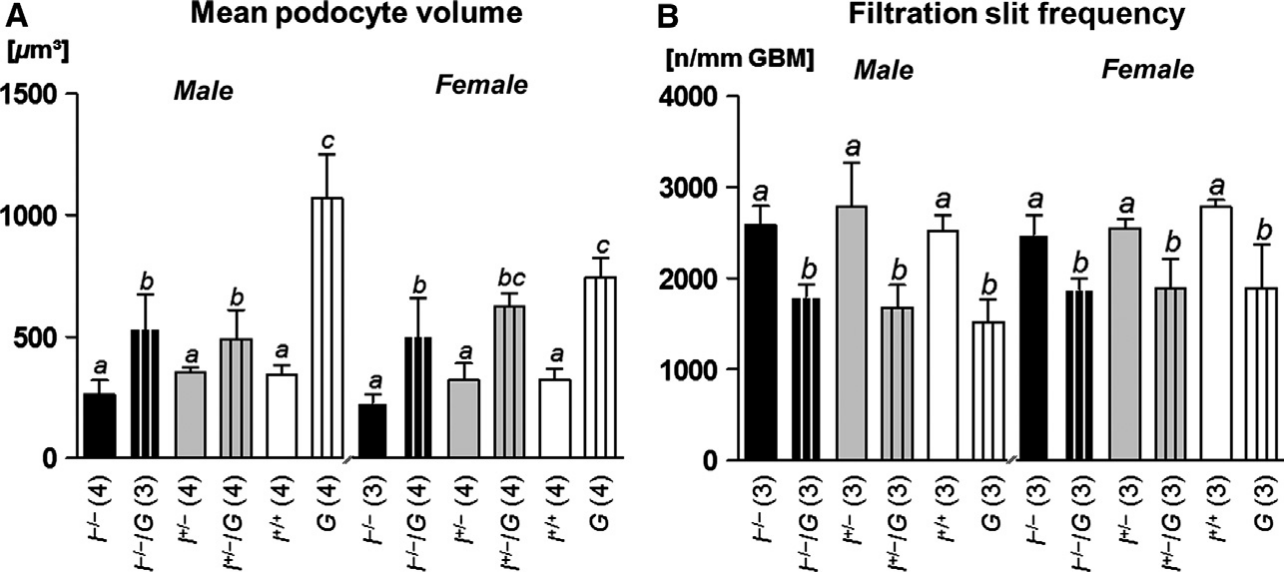
Mean podocyte volume (A) and filtration slit frequency (B) at 11 weeks of age. *I* ^*−*/*−*^/*G*, I^+/*−*^/*G*, and *G* mice display significantly higher mean podocyte volumes, as compared to *I* ^*−*/*−*^, *I* ^+/*−*^, and *I* ^*+/+*^ mice. The number of podocyte–foot process filtration slits per mm of the glomerular basement membrane (GBM) is significantly reduced in *I* ^*−*/*−*^ versus *I* ^*−*/*−*^/*G*,* I* ^+/*−*^ versus I^+/*−*^/*G*, and *I* ^*+/+*^ versus *G* mice. Data are means ± SD. One‐way ANOVA with LSD post hoc test. Statistically significant differences (*P* ≤ 0.05) are indicated by different superscripts. The numbers of examined animals are given in brackets.

Due to a significant increase of the numbers of endothelial and mesangial cells per glomerulus, the mean total numbers of cells per glomerulus in *I* ^*−/−*^ */G*,* I* ^*+/−*^ */G*, and *G* mice were significantly increased, as compared to *I* ^*−/−*^, *I* ^*+/−*^, and *I* ^*+/+*^ mice, respectively. In contrast, the mean numbers of podocytes per glomerulus were not significantly different between *I* ^*−/−*^ */G* and *I* ^*−/−*^, *I* ^*+/−*^ */G* and *I* ^*+/−*^, or *G* and *I* ^*+/+*^ mice (Fig. 6). However, the mean podocyte volume was significantly increased in GH‐transgenic mice, as compared to non‐GH‐transgenic mice of the identical *Igf1* status, except for male *I* ^*+/−*^ */G* versus *I* ^*+/−*^ mice (Fig. 7A).

#### Filtration slit frequency and glomerular basement membrane thickness

Compared to GH‐transgenic (*I* ^*−/−*^ */G*,* I* ^*+/−*^ */G* and *G*) mice, non‐GH‐transgenic mice (*I* ^*−/−*^, *I* ^*+/−*^, and *I* ^*+/+*^) exhibited significantly higher glomerular filtration slit frequencies (FSF) (Fig. 7B), whereas the true harmonic mean thickness of the glomerular basement membrane (GBM) was not significantly different in *I* ^*−/−*^ */G* versus *I* ^*−/−*^, *I* ^*+/−*^ */G* versus *I* ^*+/−*^, and *G* versus *I* ^*+/+*^ mice (Table 4).

#### Volume densities and total volumes of proximal tubular epithelial cells in the cortex and the OSM, and total numbers and mean volumes of PTE cells

**Figure 8:**
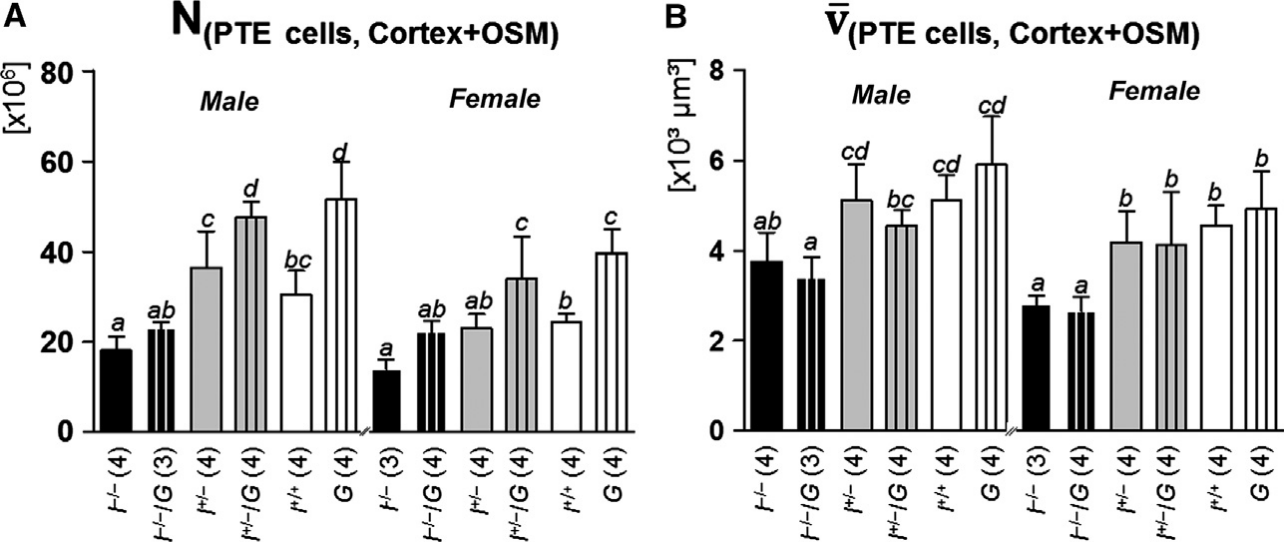
Total numbers (A) and mean volumes (B) of proximal tubular epithelial cells at 11 weeks of age. The total number of PTE cells is significantly increased in *I* ^*+/+*^ versus *G* mice and in I^+/*−*^/*G* versus *I* ^+/*−*^ mice, but not in *I* ^*−*/*−*^/*G* versus *I* ^*−*/*−*^ mice. The mean volume of PTE cells is not significantly different in *I* ^*−*/*−*^ versus *I* ^*−*/*−*^/*G*,* I* ^+/*−*^ versus I^+/*−*^/*G*, and *I* ^*+/+*^ versus *G* mice. Data are means ± SD. One‐way ANOVA with LSD post hoc test. Statistically significant differences (*P* ≤ 0.05) are indicated by different superscripts. The numbers of examined animals are given in brackets.

The volume densities of proximal tubular epithelial (PTE) cells in the renal cortex and the OSM (*V*_V(PTE/Cortex+OSM)_) did not differ significantly between *I* ^*−/−*^ */G* and *I* ^*−/−*^, *I* ^*+/−*^ */G* and *I* ^*+/−*^, or *G* and *I* ^*+/+*^ mice (Table 5). The total volume of PTE cells in the cortex and the OSM (*V*_(PTE, Cortex+OSM)_) were significantly increased in *G* versus *I* ^*+/+*^ mice. In *I* ^*−/−*^ */G* versus *I* ^*−/−*^, and in *I* ^*+/−*^ */G* versus *I* ^*+/−*^ mice the *V*_(PTE, Cortex+OSM)_ was increased, but the differences did not reach statistical relevance (Table 5). The total numbers of PTE cells in the kidneys (*N*_(PTE cells, Cortex+OSM)_) were significantly increased in *G* versus *I* ^*+/+*^ and in *I* ^*+/−*^ */G* versus *I* ^*+/−*^ mice, but not in *I* ^*−/−*^ */G* versus *I* ^*−/−*^ mice, whereas the mean volume of PTE cells ($\overset{¯}{v}$_(PTE cells, Cortex+OSM))_ was virtually unaltered between GH‐transgenic and non‐GH‐transgenic mice of corresponding *Igf1* status (Fig. 8).

**Table 5:** Quantitative stereology of renal tubules

Parameter	*n* (m/f)	*I* ^*−/−*^	*I* ^*−/−*^ */G*	*I* ^*+/−*^	*I* ^*+/−*^ */G*	*I* ^*+/+*^	*G*
		5/3	3/4	5/5	5/5	5/5	5/5
*V*_V(PT/Cortex+OSM)_	m	0.77 ± 0.03	0.74 ± 0.04	0.77 ± 0.02	0.74 ± 0.01	0.72 ± 0.02^b^	0.77 ± 0.02^b^
	f	0.78 ± 0.02^b^	0.73 ± 0.02^b^	0.78 ± 0.04^b^	0.72 ± 0.02^b^	0.74 ± 0.01	0.75 ± 0.02
*V*_(PT, Kid)_ (mm³)	m	175 ± 37	196 ± 44	456 ± 47^a^	478 ± 84^a^	352 ± 96^b^	741 ± 162^b^, ^a^
	f	101 ± 11	148 ± 8	217 ± 41^b^, ^a^	333 ± 63^b^, ^a^	261 ± 29^b^	479 ± 117^b^, ^a^
*V*_V(OT/Cortex+OSM)_	m	0.15 ± 0.02	0.16 ± 0.02	0.16 ± 0.01^a^	0.15 ± 0.01^a^	0.19 ± 0.01^b^, ^a^	0.14 ± 0.01^b^
	f	0.14 ± 0.01	0.16 ± 0.01	0.14 ± 0.03^b^, ^a^	0.17 ± 0.01^b^, ^a^	0.17 ± 0.01^a^	0.16 ± 0.01
*V*_(OT, Cortex+OSM)_ (mm³)	m	34 ± 5	41 ± 9	96 ± 16^a^	97 ± 18	93 ± 20^b^, ^a^	137 ± 30^b^, ^a^
	f	18 ± 4	33 ± 3	38 ± 3^b^, ^a^	79 ± 15^b^	59 ± 8^b^, ^a^	100 ± 23^b^, ^a^
		4/3	3/4	4/4	4/4	4/4	4/4
*V*_V(PTE/Cortex+OSM)_	m	0.29 ± 0.01	0.30 ± 0.05	0.32 ± 0.02	0.35 ± 0.06	0.33 ± 0.04	0.30 ± 0.05
	f	0.31 ± 0.04	0.29 ± 0.02	0.36 ± 0.03	0.30 ± 0.05	0.32 ± 0.01	0.29 ± 0.04
*V*_(PTE, Kid)_ (mm³)	m	68 ± 11	78 ± 6	182 ± 14^a^	216 ± 18^a^	154 ± 21^b^	304 ± 88^b^, ^a^
	f	40 ± 8	59 ± 5	95 ± 7^a^	134 ± 40^a^	111 ± 19^b^	199 ± 56^b^, ^a^
*N*_V(PTE cells/Cortex+OSM)_ (n × 10³/mm³)	m	79 ± 16^a^	90 ± 25	65 ± 15^a^	76 ± 13	65 ± 13	51 ± 4
	f	109 ± 7^a^	110 ± 17	88 ± 21^a^	77 ± 14	69 ± 4	59 ± 8

Numbers of examined animals are given in brackets. Data are means ± SD. One‐way ANOVA with LSD post hoc test.

a Statistically significant differences (*P* ≤ 0.05) between male and female mice of the identical genotype.

b Statistically significant differences (*P* ≤ 0.05) between sex‐matched *I* ^*−/−*^ versus *I* ^*−/−*^/*G*,* I* ^*+/−*^ versus I^*+/−*^/*G*, and *I* ^*+/+*^ versus *G* mice.

## Discussion

Although the formal pathogenesis of progressive glomerulosclerosis in GH‐transgenic mice has been well characterized, the exact mechanisms inducing stimulation of renal and glomerular growth, glomerular and podocyte hypertrophy, mesangial and endothelial hyperplasia, and the well‐known subsequent renal lesion patterns seen in GH‐transgenic mice are yet not fully understood. Several of the various biological actions of GH, such as stimulation of somatic growth, are indirectly mediated by GH‐induced IGF1 (Le Roith et al. 2001). Although GH stimulates the release of predominantly liver‐derived IGF1 into the circulation, elevated systemic IGF1 levels are not essential for mediation of GH‐induced IGF1‐dependent growth‐promoting actions (Yakar et al. 1999; Le Roith et al. 2001), since several, if not the majority, of the growth‐stimulating effects of IGF1 are actually mediated by locally produced IGF1, acting in an autocrine or paracrine fashion (Le Roith et al. 2001; Stratikopoulos et al. 2008). The exact roles of excess GH and of IGF1 in stimulation of renal and glomerular growth in GH‐transgenic mice, however, are still unclear. Since different glomerular cell types simultaneously express both GH and IGF1 receptors (Doi et al. 2000; Tack et al. 2002; Karl et al. 2005; Reddy et al. 2007; Vasylyeva and Ferry 2007; Bridgewater et al. 2008), a clear differentiation of excess GH and IGF1 actions promoting glomerular hypertrophy and glomerulosclerosis in vivo is complicated. Almost 25 years ago, it was demonstrated that both GH‐transgenic and *Igf1*‐transgenic mice do develop glomerular hypertrophy. However, the magnitude of glomerular enlargement displayed by *Igf1*‐transgenic mice is much lower than the glomerular hypertrophy exhibited by GH‐transgenic mice, and in contrast to GH‐transgenic mice, *Igf1*‐transgenic mice do not develop glomerulosclerosis (Doi et al. 1988, 1990). These observations indicate that both excess GH‐dependent and GH‐independent elevation of systemic IGF1 levels stimulate glomerular growth. However, stimulation of glomerular growth by systemically elevated IGF1 levels in *Igf1*‐transgenic mice without concurrently elevated systemic GH levels is obviously not sufficient to trigger the development of glomerulosclerosis (Doi et al. 1990). These findings of course do not elucidate the potential role of excess GH‐stimulated, locally produced glomerular IGF1 for the development of glomerular hypertrophy and glomerulosclerosis in GH‐transgenic mice with systemically elevated GH levels.

To dissociate the relative contributions of IGF1‐mediated and IGF1‐independent effects of GH excess on stimulation of renal growth and subsequent development of progressive glomerulosclerosis in vivo, the present study applied model‐independent stereological methods on kidney samples of a unique collective of GH‐transgenic IGF1‐deficient mice. Since permanently excessively elevated levels of GH in *G* mice induce a simultaneous increase in endogenous IGF1 levels, none of the phenotypical effects observed in *G* mice can clearly be attributed to GH or IGF1 alone by comparing *G* mice to *I* ^*+/+*^ mice. For the same reason, a comparison of *G* mice and *I* ^*−/−*^ */G* mice is also not suitable to identify IGF1‐independent effects of excess GH on renal growth and pathology. Additionally, IGF1 is a crucially important factor for embryonal and fetal renal growth and nephronogenesis, and therefore the initial “kidney status” (characterized by, e.g., kidney volume, nephron number, initial mean glomerular or podocyte volume) is not comparable between *Igf1*‐knockout mice and mice with intact *igf1* alleles. Therefore, a meaningful dissociation of IGF1‐independent effects of excess GH from GH‐induced IGF1‐mediated effects can only be performed by comparing *I* ^*−/−*^ */G* mice and *I* ^*−/−*^ mice. Correspondingly, groups of mice with identical IGF1 genotypes were compared throughout the present study (I^*−*/*−*^ vs. *I* ^*−/−*^ */G*,* I* ^*+/−*^ vs. *I* ^*+/−*^ */G*, and *I* ^*+/+*^ vs. *G*).

Implementation of studies on *Igf1*‐knockout mice is generally hindered by the low perinatal survival rates of IGF1‐deficient mice, restricting the numbers of animals available for investigation (Liu et al. 1993; Liu and LeRoith 1999; Lupu et al. 2001; Blutke et al. 2014). Although the numbers of examined *I* ^*−*/*−*^ and *I* ^*−*/*−*^/*G* mice were limited to 7–8 mice per genotype, the results of exhaustive quantitative stereological analyses were capable to clearly identify common and dissociated growth‐promoting effects of GH and IGF1 on the kidney, distinct kidney structures, and cell types.

Confirming the essential role of IGF1‐mediated GH‐stimulated postnatal somatic growth (Powell‐Braxton et al. 1993; Liu and LeRoith 1999; Le Roith et al. 2001; Blutke et al. 2014), *I* ^*−*/*−*^ and *I* ^*−*/*−*^/G mice reached only ~35% and ~57% of the body weight of sex‐matched WT control animals at 75 days of age, although the body weights of *I* ^*−*/*−*^/G mice were significantly higher than those of *I* ^*−*/*−*^ mice (Blutke et al. 2014).

In the kidneys, a disproportional stimulation of renal growth by GH‐overexpression, as indicated by increased relative kidney weights in elder (>120 days) mice of various GH‐transgenic mouse lines (Brem et al. 1989; Wanke et al. 1991), was yet not present in 75 days old GH‐transgenic mice versus non‐GH‐transgenic mice of the same *igf1* allele status. Interestingly, the effect of GH overexpression on the absolute volumes of the kidneys and of distinct kidney compartments, including the cortex, the outer stripe of the medulla (OSM), and the proximal tubules, differed in GH‐transgenic *Igf1*‐knockout mice, as compared to GH‐transgenic mice with intact *igf1* alleles. On the average, the total kidney weights and volumes of G mice were significantly increased by 86%, as compared to sex‐matched *I* ^*+/+*^ mice (93% in male mice and 78% in female mice). In contrast, the nonsignificant increase of kidney weights and volumes in *I* ^*−*/*−*^/G versus *I* ^*−*/*−*^ mice accounted merely for 37% (20% in male mice and 54% in female mice), supporting the finding that stimulation of overall renal growth by GH excess is largely but not exclusively mediated by IGF1 (Doi et al. 1990). The marked increase of the kidney volume in *G* versus *I* ^*+/+*^ mice was caused by a significant, on the average 95% increase in the volume of the renal cortex (*V*_(Cortex, Kid)_), and by a significant, on the average 78% increase in the OSM (*V*_(OSM, kid)_) in male and female *G* versus *I* ^*+/+*^ mice. The increased volumes of the renal cortex and the OSM mainly resulted from a significant, on the average 97% enlargement of the volume of proximal tubules (*V*_(PT, Cortex & OSM)_). The latter was found due to a significant increase in the total volume of the proximal tubular epithelia (PTE) in the renal cortex and the OSM. Quantitative stereological analyses revealed a hyperplastic growth of PTE cells to account for the increased total PTE volume in *G* versus *I* ^*+/+*^ mice. Although the mean cellular volume of PTE cells was virtually unaltered in all GH‐overexpressing versus non‐GH‐transgenic mice of identical *Igf1* status, the total number of PTE cells in *G* versus *I* ^*+/+*^ mice was significantly increased by 76%. In contrast, the volumes of the renal cortex, the OSM, the proximal tubules, the PTE, and the total PTE cell numbers in *I* ^*−*/*−*^/G versus *I* ^*−*/*−*^ mice and in *I* ^+/*−*^/G versus *I* ^+/*−*^ mice were throughout increased by just ~30–40%. However, due to the limited number of *Igf1*‐knockout mice of 75 days of age available for analysis, these differences did not (yet) reach the level of statistical significance. The observed differences in stimulation of tubular growth between *G*,* I* ^+/*−*^/*G*, and *I* ^*−*/*−*^/*G* mice show that IGF1 is a major mediator of PTE hyperplasia induced by GH excess, consistent with previously reported proliferative/mitogenic effects of IGF1 on proximal tubular epithelial cells in vitro (Blazer‐Yost et al. 1992).

In addition to their dwarf phenotype, and their proportionally reduced kidney sizes, the complete deficiency of IGF1 also essentially affected renal development in *I* ^*−*/*−*^ and *I* ^*−*/*−*^/*G* mice. The numbers of nephrons (*N*_(Glom,Kid)_) in the small kidneys of *I* ^*−*/*−*^ and *I* ^*−*/*−*^/*G* mice were considerably lower than in mice with intact or heterozygously deleted *igf1* alleles, while *Igf1*‐haploinsufficiency in *I* ^*+/−*^ and *I* ^*+/−*^ */G* mice did not detectably affect nephron numbers, as compared to *G* or *I* ^*+/+*^ mice. Furthermore, GH overexpression did also not significantly influence the number of nephrons in *I* ^*−/−*^ */G* versus *I* ^*−/−*^, *I* ^*+/−*^ */G* versus *I* ^*+/−*^, and *G* versus *I* ^*+/+*^ mice, indicating that GH overexpression does not affect nephronogenesis in GH transgenic mice.

In GH transgenic mice, progressive glomerular and podocyte hypertrophy, subsequent podocyte damage, and albuminuria are pathogenetic key lesions in the development of glomerulosclerosis (Wanke et al. 2001; Pavenstadt et al. 2003; Wiggins 2007). The mean glomerular volume and the mean podocyte volume, as the relevant parameters for assessment of glomerular and podocyte hypertrophy, were significantly increased in all GH‐transgenic mice versus their non‐GH‐transgenic controls of identical *Igf1* status. In parallel, mice of all investigated GH‐transgenic genotypes displayed glomerulosclerotic kidney lesions, significantly reduced podocyte filtration slit frequencies, and albuminuria. The glomerulosclerosis indices of the mice examined in the present study have already been reported in a previous publication (Blutke et al. 2014). Mice of all GH‐transgenic genotypes (*I* ^*−*/*−*^/*G*,* I* ^+/*−*^/*G*, and *G*) consistently display significantly higher glomerulosclerosis indices than non‐GH‐transgenic mice (*I* ^*−*/*−*^, *I* ^+/*−*^, and *I* ^+/+^ mice). Since glomerulosclerotic lesions were exclusively observed in GH‐transgenic mice, these lesions must be regarded as a consequence of permanently and excessively elevated levels of GH (and GH‐dependent IGF1, if present). In contrast, the elevated endogenous GH secretion with a pulsatile secretion pattern that occurs as a feedback reaction to IGF1 deficiency (Blutke et al. 2014) is apparently not sufficient to induce development of comparable glomerular alterations in (non‐GH‐transgenic) *Igf1*‐knockout mice. Since *I* ^*−/−*^ */G* mice and *I* ^*+/−*^ */G* mice display significantly lower glomerulosclerosis indices than *G* mice (Blutke et al. 2014), IGF1 deficiency apparently slightly ameliorates the severity, respectively, the velocity of progression of excess GH‐induced glomerulosclerosis in GH‐transgenic mice. On one hand, these findings indicate that excess GH‐induced IGF1 contributes to the development of glomerulosclerosis in GH‐transgenic mice. On the other hand, although the glomerulosclerosis indices in GH‐transgenic mice with intact *igf1* alleles are significantly higher than in IGF1‐deficient mice, the present data also prove that GH excess can cause glomerular and podocyte hypertrophy sufficient to induce glomerulosclerosis, independently of IGF1.

Indicating an excess GH‐stimulated glomerular growth even in the absence of IGF1, the relative mean glomerular volumes of GH‐overexpressing mice were on the average three times as large as in sex‐matched non‐GH‐transgenic mice of identical *Igf1* status, with male *G* mice exhibiting the greatest average glomerular volume increase. Distinct glomerular growth processes contributing to the enlargement of the glomerular tuft in GH‐transgenic mice with or without intact *igf1* alleles were extensively characterized by quantitative stereological analyses. Thus, glomerular hypertrophy resulted from an increased mesangial volume per glomerulus, hyperplasia of mesangial and endothelial glomerular cells, a higher glomerular capillary volume due to an increased capillary length per glomerulus, as well as from hypertrophy of podocytes. *G* mice consistently displayed the highest absolute magnitudes in the different glomerular compartment‐ and cell‐type growth parameters, whereas the relative increases in these parameters were in the same ranges in mice of all GH‐overexpressing genotypes, as compared to non‐GH‐transgenic mice of the same *Igf1* status. Therefore, the relative increase in the mean glomerular and podocyte volume in GH‐transgenic mice, as compared to non‐GH‐transgenic mice of the same *Igf1* status appears to be a critical step in the development of glomerulosclerosis in GH‐transgenic murine nephropathy models, rather than excess of a distinct absolute glomerular or podocyte size.

Podocyte hypertrophy, as an initial step in the development of podocyte damage subsequently leading to glomerulosclerotic lesions, is regarded as a common reaction of postmitotic podocytes to progressive glomerular enlargement in diverse experimental nephropathy models (Kretzler et al. 1994; Wolf and Wanke 1997; Wanke et al. 2001; Wiggins 2007). Additionally, it has been demonstrated that podocyte hypertrophy can also precede glomerular hypertrophy in murine models of diabetic nephropathy (Herbach et al. 2009) and that the glomerular podocyte is a target of direct GH action (Reddy et al. 2007; Kumar et al. 2010, 2011). Given a probable contribution of direct GH‐mediated growth stimulation to podocyte hypertrophy in the pathogenesis of glomerulosclerosis in GH‐transgenic mice, the significantly increased mean podocyte volumes in *I* ^*−*/*−*^/*G* versus *I* ^*−*/*−*^ mice indicate that the growth stimulatory effect of excess GH on podocytes can as well occur independently of IGF1. However, the exact molecular pathways of potential direct GH actions governing and modulating growth responses of different renal/glomerular cell types in the pathogenesis of glomerular hypertrophy and glomerulosclerosis will require further investigations. In parallel, the finding that the highest increases of the mean glomerular volume and of the mean podocyte volume were observed in *G* versus *I* ^*+/+*^ mice, confirms that excess GH‐induced IGF1 also substantially contributes to the development of the pathogenetically relevant alterations leading to glomerulosclerosis in GH‐transgenic mice with intact *igf1* alleles.

In summary, the data presented in this study demonstrate dissociated effects of GH and IGF1 in excess GH‐stimulated tubular and glomerular growth in vivo, and indicate that (1) IGF1 is not necessary for mediation of the effects of GH‐overabundance causing progressive glomerulosclerosis in GH‐transgenic mice; and (2) IGF1 is an important mediator of excess GH‐induced proximal tubular hyperplasia. In light of the currently “reawaken” interest in the role of GH in the pathogenesis of human kidney diseases as diabetic nephropathy, and potentially deducible therapeutic strategies for prevention and treatment of these diseases (Kumar et al. 2011), the results of the present study strongly encourage further investigations in this field.

## Conflict of Interest

None declared.

## References

[phy212709-bib-0001] Bach, L. A. , and L. J. Hale . 2015 Insulin‐like growth factors and kidney disease. Am. J. Kidney Dis. 65:327–336. 25151409 10.1053/j.ajkd.2014.05.024

[phy212709-bib-0002] Blazer‐Yost, B. L. , M. Watanabe , T. P. Haverty , and F. N. Ziyadeh . 1992 Role of insulin and IGF1 receptors in proliferation of cultured renal proximal tubule cells. Biochim. Biophys. Acta 1133:329–335. 1310625 10.1016/0167-4889(92)90055-g

[phy212709-bib-0003] Blutke, A. , M. R. Schneider , I. Renner‐Muller , N. Herbach , R. Wanke , and E. Wolf . 2014 Genetic dissection of IGF1‐dependent and ‐independent effects of permanent GH excess on postnatal growth and organ pathology of mice. Mol. Cell. Endocrinol. 394:88–98. 25017732 10.1016/j.mce.2014.07.002

[phy212709-bib-0004] Brem, G. , R. Wanke , E. Wolf , T. Buchmuller , M. Muller , B. Brenig , et al. 1989 Multiple consequences of human growth hormone expression in transgenic mice. Mol. Biol. Med. 6:531–547. 2634813

[phy212709-bib-0005] Bridgewater, D. J. , J. M. Dionne , M. J. Butt , C. L. Pin , and D. G. Matsell . 2008 The role of the type I insulin‐like growth factor receptor (IGF‐IR) in glomerular integrity. Growth Horm. IGF Res. 18:26–37. 17689124 10.1016/j.ghir.2007.06.003

[phy212709-bib-0006] Dirsch, V. M. , E. Wolf , R. Wanke , R. Schulz , W. Hermanns , and A. M. Vollmar . 1998 Effect of chronic GH overproduction on cardiac ANP expression and circulating ANP levels. Mol. Cell. Endocrinol. 144:109–118. 9863631 10.1016/s0303-7207(98)00148-8

[phy212709-bib-0007] Doi, T. , L. J. Striker , C. Quaife , F. G. Conti , R. Palmiter , R. Behringer , et al. 1988 Progressive glomerulosclerosis develops in transgenic mice chronically expressing growth hormone and growth hormone releasing factor but not in those expressing insulinlike growth factor‐1. Am. J. Pathol. 131:398–403. 3132856 PMC1880691

[phy212709-bib-0008] Doi, T. , L. J. Striker , C. C. Gibson , L. Y. Agodoa , R. L. Brinster , and G. E. Striker . 1990 Glomerular lesions in mice transgenic for growth hormone and insulinlike growth factor‐I. I. Relationship between increased glomerular size and mesangial sclerosis. Am. J. Pathol. 137:541–552. 2399934 PMC1877515

[phy212709-bib-0009] Doi, S. Q. , T. A. Jacot , D. F. Sellitti , P. Hirszel , M. H. Hirata , G. E. Striker , et al. 2000 Growth hormone increases inducible nitric oxide synthase expression in mesangial cells. J. Am. Soc. Nephrol. 11:1419–1425. 10906155 10.1681/ASN.V1181419

[phy212709-bib-0010] El‐Aouni, C. , N. Herbach , S. M. Blattner , A. Henger , M. P. Rastaldi , G. Jarad , et al. 2006 Podocyte‐specific deletion of integrin‐linked kinase results in severe glomerular basement membrane alterations and progressive glomerulosclerosis. J. Am. Soc. Nephrol. 17:1334–1344. 16611717 10.1681/ASN.2005090921

[phy212709-bib-0011] Fogo, A. , and I. Ichikawa . 1989 Evidence for the central role of glomerular growth promoters in the development of sclerosis. Semin. Nephrol. 9:329–342. 2688009

[phy212709-bib-0012] Fogo, A. , and I. Ichikawa . 1991 Evidence for a pathogenic linkage between glomerular hypertrophy and sclerosis. Am. J. Kidney Dis. 17:666–669. 2042646 10.1016/s0272-6386(12)80347-7

[phy212709-bib-0013] Grunenwald, S. , I. Tack , D. Chauveau , A. Bennet , and P. Caron . 2011 Impact of growth hormone hypersecretion on the adult human kidney. Ann. Endocrinol. (Paris) 72:485–495. 22098791 10.1016/j.ando.2011.08.001

[phy212709-bib-0014] Herbach, N. , I. Schairer , A. Blutke , S. Kautz , A. Siebert , B. Goke , et al. 2009 Diabetic kidney lesions of GIPRdn transgenic mice: podocyte hypertrophy and thickening of the GBM precede glomerular hypertrophy and glomerulosclerosis. Am. J. Physiol. Renal. Physiol. 296:F819–F829. 19211686 10.1152/ajprenal.90665.2008

[phy212709-bib-0015] Herbach, N. , M. Bergmayr , B. Goke , E. Wolf , and R. Wanke . 2011 Postnatal development of numbers and mean sizes of pancreatic islets and beta‐cells in healthy mice and GIPR(dn) transgenic diabetic mice. PLoS ONE 6:e22814. 21818396 10.1371/journal.pone.0022814 PMC3144241

[phy212709-bib-0016] Hermanns, W. , K. Liebig , and L. C. Schulz . 1981 Postembedding immunohistochemical demonstration of antigen in experimental polyarthritis using plastic embedded whole joints. Histochemistry 73:439–446. 7035413 10.1007/BF00495658

[phy212709-bib-0017] Hoeflich, A. , S. Nedbal , W. F. Blum , M. Erhard , H. Lahm , G. Brem , et al. 2001 Growth inhibition in giant growth hormone transgenic mice by overexpression of insulin‐like growth factor‐binding protein‐2. Endocrinology 142:1889–1898. 11316754 10.1210/endo.142.5.8149

[phy212709-bib-0018] Hoeflich, A. , M. M. Weber , T. Fisch , S. Nedbal , C. Fottner , M. W. Elmlinger , et al. 2002 Insulin‐like growth factor binding protein 2 (IGFBP‐2) separates hypertrophic and hyperplastic effects of growth hormone (GH)/IGF‐I excess on adrenocortical cells in vivo. FASEB J. 16:1721–1731. 12409314 10.1096/fj.02-0349com

[phy212709-bib-0019] Howard, V. , and M. G. Reed . 2004 Unbiased stereology. Garland Science/BIOS Scientific Publishers, New York.

[phy212709-bib-0020] Kamenicky, P. , G. Mazziotti , M. Lombes , A. Giustina , and P. Chanson . 2014 Growth hormone, insulin‐like growth factor‐1, and the kidney: pathophysiological and clinical implications. Endocr. Rev. 35:234–281. 24423979 10.1210/er.2013-1071

[phy212709-bib-0021] Karl, M. , M. Potier , I. H. Schulman , A. Rivera , H. Werner , A. Fornoni , et al. 2005 Autocrine activation of the local insulin‐like growth factor I system is up‐regulated by estrogen receptor (ER)‐independent estrogen actions and accounts for decreased ER expression in type 2 diabetic mesangial cells. Endocrinology 146:889–900. 15550505 10.1210/en.2004-1121

[phy212709-bib-0022] Klahr, S. , G. Schreiner , and I. Ichikawa . 1988 The progression of renal disease. N. Engl. J. Med. 318:1657–1666. 3287163 10.1056/NEJM198806233182505

[phy212709-bib-0023] Kretzler, M. , I. Koeppen‐Hagemann , and W. Kriz . 1994 Podocyte damage is a critical step in the development of glomerulosclerosis in the uninephrectomised‐desoxycorticosterone hypertensive rat. Virchows Arch. 425:181–193. 7952502 10.1007/BF00230355

[phy212709-bib-0024] Kriz, W. 1996 Progressive renal failure–inability of podocytes to replicate and the consequences for development of glomerulosclerosis. Nephrol. Dial. Transplant. 11:1738–1742. 8918614

[phy212709-bib-0025] Kriz, W. 2002 Podocyte is the major culprit accounting for the progression of chronic renal disease. Microsc. Res. Tech. 57:189–195. 12012382 10.1002/jemt.10072

[phy212709-bib-0026] Kriz, W. 2012 Glomerular diseases: podocyte hypertrophy mismatch and glomerular disease. Nat. Rev. Nephrol. 8:618–619. 23007616 10.1038/nrneph.2012.198

[phy212709-bib-0027] Kriz, W. , and L. Bankir . 1988 A standard nomenclature for structure of the kidney. The Renal Commission of the International Union of Physiological Sciences(IUPS). Anat. Embryol. (Berl) 178:N1–N8. 3394952

[phy212709-bib-0028] Kriz, W. , and H. Koepsell . 1974 The structural organization of the mouse kidney. Z. Anat. Entwicklungsgesch. 144:137–163. 4472393 10.1007/BF00519771

[phy212709-bib-0029] Kriz, W. , and M. LeHir . 2005 Pathways to nephron loss starting from glomerular diseases‐insights from animal models. Kidney Int. 67:404–419. 15673288 10.1111/j.1523-1755.2005.67097.x

[phy212709-bib-0030] Kriz, W. , M. Kretzler , M. Nagata , A. P. Provoost , I. Shirato , S. Uiker , et al. 1996 A frequent pathway to glomerulosclerosis: deterioration of tuft architecture‐podocyte damage‐segmental sclerosis. Kidney Blood Press. Res. 19:245–253. 8956236 10.1159/000174083

[phy212709-bib-0031] Kumar, P. A. , K. Kotlyarevska , P. Dejkhmaron , G. R. Reddy , C. Lu , M. S. Bhojani , et al. 2010 Growth hormone (GH)‐dependent expression of a natural antisense transcript induces zinc finger E‐box‐binding homeobox 2 (ZEB2) in the glomerular podocyte: a novel action of gh with implications for the pathogenesis of diabetic nephropathy. J. Biol. Chem. 285:31148–31156. 20682777 10.1074/jbc.M110.132332 PMC2951188

[phy212709-bib-0032] Kumar, P. A. , F. C. Brosius 3rd , and R. K. Menon . 2011 The glomerular podocyte as a target of growth hormone action: implications for the pathogenesis of diabetic nephropathy. Curr. Diabetes Rev. 7:50–55. 21067510 10.2174/157339911794273900 PMC4007067

[phy212709-bib-0033] Le Roith, D. , C. Bondy , S. Yakar , J. L. Liu , and A. Butler . 2001 The somatomedin hypothesis: 2001. Endocr. Rev. 22:53–74. 11159816 10.1210/edrv.22.1.0419

[phy212709-bib-0034] Liu, J. L. , and D. LeRoith . 1999 Insulin‐like growth factor I is essential for postnatal growth in response to growth hormone. Endocrinology 140:5178–5184. 10537147 10.1210/endo.140.11.7151

[phy212709-bib-0035] Liu, J. P. , J. Baker , A. S. Perkins , E. J. Robertson , and A. Efstratiadis . 1993 Mice carrying null mutations of the genes encoding insulin‐like growth factor I (Igf‐1) and type 1 IGF receptor (Igf1r). Cell 75:59–72. 8402901

[phy212709-bib-0036] Lupu, F. , J. D. Terwilliger , K. Lee , G. V. Segre , and A. Efstratiadis . 2001 Roles of growth hormone and insulin‐like growth factor 1 in mouse postnatal growth. Dev. Biol. 229:141–162. 11133160 10.1006/dbio.2000.9975

[phy212709-bib-0037] Mak, R. H. , W. W. Cheung , and C. T. Roberts Jr . 2008 The growth hormone‐insulin‐like growth factor‐I axis in chronic kidney disease. Growth Horm. IGF Res. 18:17–25. 17826224 10.1016/j.ghir.2007.07.009 PMC2706146

[phy212709-bib-0038] Miquet, J. G. , L. Gonzalez , M. N. Matos , C. E. Hansen , A. Louis , A. Bartke , et al. 2008 Transgenic mice overexpressing GH exhibit hepatic upregulation of GH‐signaling mediators involved in cell proliferation. J. Endocrinol. 198:317–330. 18480380 10.1677/JOE-08-0002

[phy212709-bib-0039] el Nahas, A. M. 1989 Glomerulosclerosis: insights into pathogenesis and treatment. Nephrol. Dial. Transplant. 843–853. 10.1093/ndt/4.10.843 2515487

[phy212709-bib-0040] Norstedt, G. , and R. Palmiter . 1984 Secretory rhythm of growth hormone regulates sexual differentiation of mouse liver. Cell 36:805–812. 6323022 10.1016/0092-8674(84)90030-8

[phy212709-bib-0041] Nyengaard, J. R. 1999 Stereologic methods and their application in kidney research. J. Am. Soc. Nephrol. 10:1100–1123. 10232698 10.1681/ASN.V1051100

[phy212709-bib-0042] Pavenstadt, H. , W. Kriz , and M. Kretzler . 2003 Cell biology of the glomerular podocyte. Physiol. Rev. 83:253–307. 12506131 10.1152/physrev.00020.2002

[phy212709-bib-0043] Powell‐Braxton, L. , P. Hollingshead , C. Warburton , M. Dowd , S. Pitts‐Meek , D. Dalton , et al. 1993 IGF‐I is required for normal embryonic growth in mice. Genes Dev. 7:2609–2617. 8276243 10.1101/gad.7.12b.2609

[phy212709-bib-0044] Ramage, I. J. , A. G. Howatson , J. H. McColl , H. Maxwell , A. V. Murphy , and T. J. Beattie . 2002 Glomerular basement membrane thickness in children: a stereologic assessment. Kidney Int. 62:895–900. 12164871 10.1046/j.1523-1755.2002.00527.x

[phy212709-bib-0045] Reddy, G. R. , M. J. Pushpanathan , R. F. Ransom , L. B. Holzman , F. C. Brosius 3rd , M. Diakonova , et al. 2007 Identification of the glomerular podocyte as a target for growth hormone action. Endocrinology 148:2045–2055. 17272398 10.1210/en.2006-1285

[phy212709-bib-0046] Scherle, W. 1970 A simple method for volumetry of organs in quantitative stereology. Mikroskopie 26:57–60. 5530651

[phy212709-bib-0047] Schrijvers, B. F. , A. S. De Vriese , and A. Flyvbjerg . 2004 From hyperglycemia to diabetic kidney disease: the role of metabolic, hemodynamic, intracellular factors and growth factors/cytokines. Endocr. Rev. 25:971–1010. 15583025 10.1210/er.2003-0018

[phy212709-bib-0048] Stefaneanu, L. , K. Kovacs , A. Bartke , A. Mayerhofer , and T. E. Wagner . 1993 Pituitary morphology of transgenic mice expressing bovine growth hormone. Lab. Invest. 68:584–591. 8388524

[phy212709-bib-0049] Sterio, D. C. 1984 The unbiased estimation of number and sizes of arbitrary particles using the disector. J. Microsc. 127–136. 10.1111/j.1365-2818.1984.tb02501.x 6737468

[phy212709-bib-0050] Stratikopoulos, E. , M. Szabolcs , I. Dragatsis , A. Klinakis , and A. Efstratiadis . 2008 The hormonal action of IGF1 in postnatal mouse growth. Proc. Natl. Acad. Sci. U. S. A. 105:19378–19383. 19033454 10.1073/pnas.0809223105 PMC2614769

[phy212709-bib-0051] Tack, I. , S. J. Elliot , M. Potier , A. Rivera , G. E. Striker , and L. J. Striker . 2002 Autocrine activation of the IGF‐I signaling pathway in mesangial cells isolated from diabetic NOD mice. Diabetes 51:182–188. 11756339 10.2337/diabetes.51.1.182

[phy212709-bib-0052] Thrailkill, K. M. , R. Clay Bunn , and J. L. Fowlkes . 2009 Matrix metalloproteinases: their potential role in the pathogenesis of diabetic nephropathy. Endocrine 35:1–10. 18972226 10.1007/s12020-008-9114-6 PMC2629499

[phy212709-bib-0053] Vasylyeva, T. L. , and R. J. Ferry Jr . 2007 Novel roles of the IGF‐IGFBP axis in etiopathophysiology of diabetic nephropathy. Diabetes Res. Clin. Pract. 76:177–186. 17011663 10.1016/j.diabres.2006.09.012 PMC1892792

[phy212709-bib-0054] von Waldthausen, D. C. , M. R. Schneider , I. Renner‐Muller , D. N. Rauleder , N. Herbach , B. Aigner , et al. 2008 Systemic overexpression of growth hormone (GH) in transgenic FVB/N inbred mice: an optimized model for holistic studies of molecular mechanisms underlying GH‐induced kidney pathology. Transgenic Res. 17:479–488. 18097769 10.1007/s11248-007-9163-2

[phy212709-bib-0055] Wanke, R. , W. Hermanns , S. Folger , E. Wolf , and G. Brem . 1991 Accelerated growth and visceral lesions in transgenic mice expressing foreign genes of the growth hormone family: an overview. Pediatr. Nephrol. 5:513–521. 1911131 10.1007/BF01453693

[phy212709-bib-0056] Wanke, R. , E. Wolf , W. Hermanns , S. Folger , T. Buchmuller , and G. Brem . 1992 The GH‐transgenic mouse as an experimental model for growth research: clinical and pathological studies. Horm. Res. 37(Suppl. 3):74–87. 1427647 10.1159/000182406

[phy212709-bib-0057] Wanke, R. , E. Wolf , G. Brem , and W. Hermanns . 1996 Physiology and pathology of growth — studies in GH transgenic mice. J. Anim. Breed. Genet. 113:445–456.

[phy212709-bib-0058] Wanke, R. , E. Wolf , G. Brem , and W. Hermanns . 2001 Role of podocyte damage in the pathogenesis of glomerulosclerosis and tubulointerstitial lesions: findings in the growth hormone transgenic mouse model of progressive nephropathy. Verh. Dtsch. Ges. Pathol. 85:250–256. 11894406

[phy212709-bib-0059] Weibel, E. R. 1979 Stereological methods I. Practical methods for biological morphometry. Academic press, London.

[phy212709-bib-0060] Wiggins, R. C. 2007 The spectrum of podocytopathies: a unifying view of glomerular diseases. Kidney Int. 71:1205–1214. 17410103 10.1038/sj.ki.5002222

[phy212709-bib-0061] Wolf, E. , and R. Wanke . 1997 Growth hormone overproduction in transgenic mice: phenotypic alterations and deduced animal models. Springer Verlag, Berlin.

[phy212709-bib-0062] Wolf, E. , E. Kahnt , J. Ehrlein , W. Hermanns , G. Brem , and R. Wanke . 1993 Effects of long‐term elevated serum levels of growth hormone on life expectancy of mice: lessons from transgenic animal models. Mech. Ageing Dev. 68:71–87. 8350664 10.1016/0047-6374(93)90141-d

[phy212709-bib-0063] Wolf, E. , H. Lahm , M. Wu , R. Wanke , and A. Hoeflich . 2000 Effects of IGFBP‐2 overexpression in vitro and in vivo. Pediatr. Nephrol. 14:572–578. 10912521 10.1007/s004670000362

[phy212709-bib-0064] Yakar, S. , J. L. Liu , B. Stannard , A. Butler , D. Accili , B. Sauer , et al. 1999 Normal growth and development in the absence of hepatic insulin‐like growth factor I. Proc. Natl. Acad. Sci. U. S. A. 96:7324–7329. 10377413 10.1073/pnas.96.13.7324 PMC22084

[phy212709-bib-0065] Yang, C. W. , L. J. Striker , J. J. Kopchick , W. Y. Chen , C. M. Pesce , E. P. Peten , et al. 1993 Glomerulosclerosis in mice transgenic for native or mutated bovine growth hormone gene. Kidney Int. Suppl. 39:S90–S94. 8468934

